# Characterization of sacha inchi (Plukenetia volubilis) and taro (Colocasia esculenta) flours with potential application in the preparation of both gluten-free and high protein foods.

**DOI:** 10.12688/f1000research.130394.1

**Published:** 2023-04-06

**Authors:** Ruby Aracely Narváez Cadena, Angie Paola Salas Zambrano, Jesús Eduardo Bravo Gómez, Karen Sofia Muñoz Pabon, Diego Fernando Roa-Acosta

**Affiliations:** 1 Departamento de Agroindustria, Universidad del Cauca, Popayan, Colombia

**Keywords:** Rheology properties, Proximal composition, Microbiological quality, Gluten-free foods

## Abstract

**Background:**Interest in alternative sources of tubers and legumes has increased in recent years because of the constant search for raw materials that provide bioactive compounds with antioxidant potential benefits for consumers. The functionality of new raw materials is sought through physical and/or chemical modifications to develop and innovate new foods. The objective of this study was to characterize taro (TF) (Colocasia esculenta) and sacha inchi (Plukenetia volubilis) flours, obtained by the wet (SIF-WM) and defatted method (SIF-DM), as an alternative for the formulation of new functional foods.

**Methods:**The free polyphenols of the different mixtures were analyzed, and the antioxidant properties of the extracts obtained were measured using ABTS ^.*^ (2,2′-Azino-bis(3-ethylbenzthiazoline-6-sulfonic acid). The ABTS radical method, which reacts with the phenolic compounds of the food matrix, using Trolox as a standard.

The blends were subjected to pasting analysis, flow profile tests, determination of viscoelastic properties (temperature sweep). Characterization of common microorganisms in these foods was performed.

**Results:** The highest protein value was obtained in the sacha inchi flour obtained by the defatted method (72.62). The majority of components in taro were carbohydrates (85.4%). About antioxidant and determination of free polyphenols, taro flour obtained values of 2.71 µmol ET/g and 7.47 mg EAG/g, higher than Sacha inchi flours. In the rheological analysis (pasting properties, flow profile, and viscoelasticity), we observed that adding taro flour in different mixtures increases the viscosity peak and a lower breakdown, while there was an increase in setback. Except for defatted sacha flour, the others presented a flow index >1 before heating, showing a dilatant fluid behavior. The presence of Taro flour improves gel formation and stability. *Staphylococcus aureus* and *Salmonella* were present in the taro flour.

**Conclusions:** The flours analyzed represent a raw material with great potential for the development of gluten-free foods with functional properties.

## Introduction

According to Ref. 1, in 2050, if economic growth is low, governance performance is weak and food demand is very high, driven by rapid population growth, a high intensity of land use will be required, leading to an expansion of crops to meet demand, under these conditions food insecurity is likely to worsen, especially in developing regions. Improved access to resources and a growing population can generate a high demand for food, especially for meat and dairy products. ^2^ These changes in livestock production are environmentally unsustainable, due to the demand for resources such as the use of land to produce crops needed for animal feed and fresh water; furthermore, animal production is associated with the generation of greenhouse gases, a situation that contributes to the acceleration of global warming. ^3^ The consumption of meat and dairy products are important sources of protein, vitamins, and minerals in the human diet. ^2^

In this scenario, we must design food systems to contribute to global environmental sustainability and meet nutritional needs, proposing diets based on plants that allow including adjustments according to each region coupled with cultural customs. ^4^ Underutilized or orphan crops are used to design original foods, not aimed at international trade, however, because of their high adaptation to the local environment, these crops play an important role in regional nutritional security. ^5^ The mixture of plants such as cereals, pseudocereals, legumes, tubers, among others, are used to create different products with adequate balance of protein, carbohydrates, fiber and micronutrients such as vitamins and minerals.

For example, Sacha inchi ( *Plukenetia volubilis*) known as wild peanut, Inca peanut, sacha peanut or mountain peanut, is an oleaginous plant that belongs to the Euphorbiaceae family. ^6^ Today, Sacha inchi is still cultivated in the lowlands of the Peruvian Amazon and has been planted for centuries by the indigenous population. ^7^ In Colombia, according to Ref. 8, production exceeds 2,400 tons of Sacha Inchi seed. The department of Putumayo is the largest producer of sacha inchi with 282 hectares, followed by Valle del Cauca, Caquetá and Antioquia.

Sacha inchi is a promising and industrializable food native to the Amazon that has essential unsaturated fatty acids such as omega-3, as alpha-linoleic acid with 47.7% to 51.9% and omega-9 as oleic acid with 7.9 to 8.9% by weight of oil, 27.4% protein, 4% ash and about 50% oil. ^9^ These nutritional properties make sacha a favorable food for health and a suitable crop for developing high-protein and gluten-free foods.

Taro is a tuber abundant in starch of which 17-28% is amylase and the remaining is amylopectin. ^10^ Taro has a high content of resistant starch that allows slow digestion with valuable effects on cholesterol and blood glucose regulation. ^11^ Taro has a high carbohydrate (59.36%) and protein (24.99%) content, the mineral content of Taro, the mineral present in the highest amount is magnesium 242.373 mg/kg, followed by calcium 94.455 mg/kg, iron 8.351 mg/kg and zinc 6.210 mg/kg, and vitamin C 0.188 mg/100 mg, vitamin B1 0.047 mg/100 mg, and Vitamin B3 0.078 mg/100 mg. ^12^ Compared to other tubers such as sweet potato, potato, cassava and yam; taro has a higher protein and fat content. ^12^ Likewise, Taro has flavonoids and phenolic acids that have antioxidant properties; flavonoids, the largest group of phenolic compounds identified in the whole plant, are associated with reducing many degenerative diseases. ^11^

Foodstuffs are complex systems of great nutritional richness and therefore sensitive to attack by microorganisms (bacteria, fungi and yeasts). The main mode of contamination of raw materials is animal defecation, manure fertilization and recontamination persisting in facilities and transport; in addition, insects and rodents are a source of contamination. ^13^ The low water activity (a _w_ < 0.60) of meal does not favor microbial growth; however, contaminating spores along with inactive microorganisms will remain viable for prolonged periods and constitute a potential health hazard. As a persistence mechanism, *Salmonella* and other pathogens form biofilms that protect against disinfection and increase their tolerance to drying processes. ^13^

In food matrices there is always a microbial load that must not exceed certain limits, according to Colombian regulations, which must be controlled to avoid the deterioration of the product and the consequent loss of its quality and suitability for consumption.

The design and production of new foods that respond to food and nutritional security amid climate change in low-income countries is a challenging issue, so this study aimed to characterize the chemical, microbiological, rheological and bioactive properties of Taro and sacha inchi flours as potential foods applied in the formulation of different foods.

## Methods

The taro tubers were bought in the Municipality of Orito Putumayo, located at 0° 38′ North Latitude and 76° 37′ West Latitude of Greenwich. Average temperature of 25°C and relative humidity of 88%. The fresh Sacha inchi almonds were supplied by the company Fruty Amazónicos S.A.S. located in the village of La Concordia in the municipality of Valle del Guamuez, located at 00° 25″ north latitude and 76° 54″ west longitude. Temperatures range between 27°C and 40°C.

### Obtaining taro flour

The taro flour (TF) was obtained following the methodology of Ref. 10. The raw material was received, the peel was removed; it was washed to eliminate impurities and then the edible part was cut into slices to facilitate dehydration; then it was weighed on a FENIX Electronic Weight Only Table Scale to verify yield, subsequently it was dried in a rotary oven (ORVES, Colombia) at a temperature of 60°C for five hours to eliminate excess moisture. The dried slices were then ground in an electric mill (Quaker City Mill, model 4-e, Philadelphia) and then passed through a 30-mesh sieve system to achieve homogeneity in flour particle size.

### Obtaining Sacha inchi flour

Sacha inchi seeds were received in white kernel, *i.e.,* without shell or husk. The flour was obtained using the two methods described below:

*Wet method*

Sacha inchi flour from the wet method (SIF-WM), was obtained as follows: wet milling was performed in a blender (Oster, BLST 4655, Colombia) of 1.25 L, to which the sacha inchi and water were added in a 1:3 ratio, this, in order to disintegrate the grain. Obtaining a milky and homogeneous suspension. In order to separate the insoluble extract (cake) from the water-soluble extract (milk), a cloth filter was used and then the cake was dried in an oven at a temperature of 65°C for 3 hours. Once dehydrated, it was immediately packed in a hermetically sealed polyethylene bag. ^14^

*Defatted method*

The sacha inchi flour from the defatted method (SIF-DM) was obtained as follows: the Sacha inchi kernel was placed in the hopper of the automatic tactile oil extraction press (CGLDENWALL, store model K28, Shanghai China), a temperature of 124°C was used. The press used the screw, which pushed the material into the main pressing cylinder, the pitch of the screw and the depth of the spiral were reduced, decreasing the volume available in the chamber, and the material being subjected to high pressure accompanied by friction against the closed bottom wall of the cylinder produced the extraction of the oil. The oil then flows through the existing orifices in the cylinder. The remaining material exits through the pressing system and the cake outlet nozzle. ^15^

### Proximate analysis

The proximate composition (protein, lipids, dietary fiber, ash and moisture) of taro and sacha inchi flours was determined according to the methods proposed by the AOAC (Association of Official Analytical Chemists, 1990) ^16^ and the carbohydrate content was estimated by difference. The details of the method are explained in more detail in the protocols uploaded in the repository.

*Water activity measurement*

To determine the a _w_ of the raw materials, the powdered sample was placed in a Decagon Pawkit portable water activity meter until the cuvette was covered. Before reporting the measurement, the equipment was calibrated with standard salts at 0.25 (13.41 mol/kg LiCl 0.25 a _w_) and 0.76 (6.00 mol/kg NaCl 0.76 a _w_).

*Protein*

On nitrogen-free paper, 0.2 to 0.8 g of sample plus 1 g of kjeldahl catalyst were weighed and placed in digestion tubes. 10 mL of sulfuric acid were added. Then, gentle heating was initiated until no foaming or splashing was observed, using a temperature ramp as follows: 125°C for 30 min, 270°C for 30 min and 400°C for 140 min.

The samples were digested until they were completely clear and translucent, free of organic matter, in the laboratory Kjeldahl digester (Raypa MBC-6/N, Spain).

Then they were cooled to room temperature in the Raypa distillation unit, after which each sample was titrated with 0.1 N HCl.

*Lipids*

For lipid testing, 1 g of sample was weighed in the extraction cartridges, then 80 mL of petroleum ether was added, immediately transferred in the rack to the laboratory Soxhlet and Randall Extractor (SX-6MP, RAYPA, Spain). After the extraction time and solvent recovery, the samples were placed in an oven at 60°C for 1 hour to eliminate the remaining ether.

*Fiber*

For fiber testing, 1-2 g of the degreased sample were transferred to the laboratory fiber extractor (F-6P Fibertest, Spain) and fixed to the angle of the front part of the unit. Then 150 mL of 0.255 N H _2_SO _4_ was heated in an Erlenmeyer flask, when it was boiling, it was placed on top of the coolant, the heating knob was adjusted to boiling point 3 or 4 and left boiling for 30 minutes. At the end of this time, it was filtered and washed with distilled water and the operation was repeated 3 times using 30 mL of water each time. Then, 150 mL of sodium hydroxide solution preheated to 90°C was placed in the upper part of the cooler; it was brought to boiling and kept for another 30 minutes. Then it was filtered and washed three times with boiling water. The sample was then placed in an oven at a temperature of 100-110°C until a constant weight was obtained.

### Determination of free polyphenols, FPP fraction

The determination of polyphenols was performed according to Ref. 17, with slight modifications. Two grams of the sample were weighed in a 50 mL falcon tube. The first fraction was added to the sample 8 mL of solution with 80% ethanol and 20% water and 1% formic acid (80 mL ethanol + 20 mL water + 1 mL formic acid). The samples were shaken for 25 minutes in a shaker at 200 RPM at room temperature. The samples were then centrifuged at 3500 rpm for 5 minutes at room temperature. Subsequently, the supernatant was taken in a new falcon tube (50 mL) and then 40 microliters of EDTA 2% was added to the supernatant.

For the second extraction, 8 mL of 70% acetone with 1% formic acid (70 mL acetone + 30 mL water + 1 mL formic acid) was added to the pellet of the first extraction. The pellet was shaken in solution for 25 minutes in the shaker at 200 RPM at room temperature. Subsequently, the samples were centrifuged at 3500 rpm for 5 minutes at room temperature and the second supernatant was poured into the first supernatant and mixed. Finally, the supernatant mixture was made up to 20 mL using distilled water and the absorbance was measured in the spectrophotometer (Thermo scientific, Genesys 10S UV VIS).

### Determination of antioxidant activity by the ABTS method

In a test tube 4 mL of ABTS (2,2′-Azino-bis(3-ethylbenzthiazoline-6-sulfonic acid), solution was added and to start the reaction 135 μL standard solution and vortexed for 5 seconds. A reagent blank was carried with 4 mL of 4.5 acetate buffer and 135 μL of ethanol, point 0 was prepared by adding 4 mL of ABTS solution and 135 μL of ethanol. Finally, the tube was capped and waited exactly 30 minutes to measure the absorbance in the spectrophotometer (Thermo scientific, Genesys 10S UV VIS) at a wavelength of 729 nm.

### Rheological analysis

*Pasting properties*

The pasting properties of each dispersion were determined using a rheometer (TA INSTRUMENTS, AR 1500, New Castel, USA), equipped with a starch pasting cell. Then, a suspension of flour in water with a concentration of 12% (w/w) in 25 g was prepared and exposed to heating and cooling. The following samples were analyzed: 100% TF; 100% SIF-DM; 100% SIF-WM; 25% SIF-DM: 75% TF; 50% SIF-DM: 75% TF; 75% SIF-DM: 25% TF; 25% SIF-WM: 75% TF; 50% SIF-WM: 75% TF; 75% SIF-WM: 25% TF. The shear rate was kept constant at 16.75 s ^-1^, throughout the heating and cooling range (25°C-90°C-25°C) while the heating rate was 10°C/min. The following parameters were obtained from the rheological analysis maximum viscosity [Pa.s]; minimum viscosity [Pa.s]; breakthrough viscosity [°C] and setback viscosity [Pa.s]. Finally, using the Savistky-Golay function, the data were smoothed in the GraphPad Prism 8.0.1 program (RRID:SCR_002798). ^18^^,^^19^

*Flow profile*

Flow properties were performed according to the methodology of Ref. 18 with modifications. An AR1500 rheometer (TA Instruments, New Castel, USA) was used in this study. The average viscosity was determined at 25°C for 12 min with a shear rate increasing in 4 steps as follows:

Step 1: 1×10 ^-3^ s ^-1^ to 1×10 ^-2^ s ^-1^

Step 2: 0.01 s ^-1^ to 0.1 s ^-1^

Step 3: 0.1 s ^-1^ to 1 s ^-1^

Step 4: 1 s ^-1^ to 100 s ^-1^

Subsequently, the sample was heated to 90°C. Once this process was completed, the average viscosity was determined again at 25°C, subjecting the sample to the same shear rate.

Flow curves (shear stress versus shear rate) were obtained and fitted to the power law model shown in Equation 1 *.*

$$T=K*y^{n}$$ (1)

Where:

T is the shear stress (Pa).

*y* is the shear velocity (s ^-1^)

*K* is the coefficient of consistency (Pa. s ^-1^)

*n* is the flow behavior index

The flow behavior index indicates Newtonian flow behavior when *n* = 1, shear thinning behavior when *n* < 1 and shear thickening when *n* > 1). Consistency and creep were determined before and after heating in order to determine the effect of thermal processing on these parameters.

*Determination of viscoelastic properties (temperature sweep)*

For the determination of viscoelastic properties (temperature sweep) the method of Ref. 20 was used, the viscoelastic properties were determined in a rheometer (TA INSTRUMENTS, AR 1500, New Castel, USA) using a system of parallel flat plates, of 40 mm diameter and 1500 μm distance between plates. The edges of the plates were sealed with petroleum jelly, with the purpose of controlling evaporation and avoiding variations in concentrations of the aqueous suspensions used.

The samples were subjected to a cycle of dynamic heating (25-85°C), stabilization (85°C for 2 minutes) and cooling (85-25°C) at 10°C/min, with a frequency of 0.5 Hz and 0.5% deformation. The profiles of the viscoelastic moduli as a function of temperature were recorded using the equipment’s software.

### Microbiological characterization

The microbiological characterization of the flours was carried out, considering the methodology of a previous publication by the authors reported in Ref. 21, which was based on parameters established by the Colombian technical standard NTC that applies for each microorganism.

To perform the microbiological characterization, 10 g of sample were taken in triplicate, then diluted in 90 mL of peptonized water and mixed at 150 rpm for 10 min in a shaker (MaxQ 4450 orbital Thermo Ficher Scientific USA), following this, for each microorganism the seeding of the appropriate dilution and selective medium is described. The details of the method are explained in more thoroughly in the protocols uploaded in the repository https://doi.org/10.5281/zenodo.7582202.

*Coliforms*

1 mL of each replicate was taken and seeded in a previously sterile Petri dish by seeding in depth on chromogenic colinstant agar for 24-48 h of incubation at 35°C.

*Mesophiles*

1 mL of sample was taken from each replicate, and each dilution was seeded by immersion in previously sterile Petri dishes in Plate Count Agar (PCA) agar for 24-72 h at 30°C.

*Fungi and yeasts*

100 μL of each replicate was taken and seeded per surface in previously sterile Petri dish by surface plate seeding on Potato Dextrose Agar (PDA) agar for 24-72 h incubation at 30°.

*Bacillus cereus*

100 μL of each replicate was taken and surface seeded in previously sterile Petri dish by surface plate seeding on Mannitol egg Yolk Polymyxin agar (MYP) agar with egg yolk specific for *Bacillus cereus* for 24 h incubation at 37°C.

*Staphylococcus aureus*

100 μL were taken from each replicate and seeded per surface in a previously sterile Petri dish, by surface plate counting on Baird Parker agar at 24 h incubation at 35°C, and then incubated for 24 h at 35°C.

*Salmonella*

25 g of sample were taken in duplicate, then diluted in 225 mL of buffered pepton water and mixed at 150 rpm for 10 min in shaker (MaxQ 4450 orbital Thermo Ficher Scientific USA), once this time was over, they were left in incubation at 37°C for 18 h, after which 100 μL were inoculated in 10 mL of Rappaport Vassiliadis malachite green broth (RVS medium) at 41. Furthermore, 1000 μL were inoculated in 10 mL of Tetrationate Mueller Kauffmann broth (MKTTn medium) at 37°C for 24 h. After this time, the surface of a Petri dish containing XLD agar selective medium was seeded by means of a loop in such a way that asylated colonies were obtained, Salmonella Shiguella agar was incubated at 34°C for 24 h, at the end of which typical colonies of *Salmonella* spp. were observed.

### Statistical analysis

A completely randomized design was used to evaluate the rheological properties of native and formulated flours. The design was based on the type of flour and its inclusion levels in the formulated flours. The three inclusion levels were 25%, 50%, 75%. The response variables were viscosity as a function of temperature-shear rate and viscoelastic modulus (G′ and G″).

The results were presented as the mean ± standard deviation of triplicate experiments. One-way analysis of variance (ANOVA) was used to compare means. Differences between means were considered significant at P < 0.05 using Tukey’s new multiple range test. Data were subjected to analysis with Minitab version 20 (RRID:SCR_014483). Graphs were generated in Graphpad Prism 5.0 (RRID:SCR_02798).

Both statistical analysis and graph generation can be completed with Graphpad Prism 5.0. There are some open-source software that could be used to perform similar functions: Oracle SQL and Python (RRID:SCR_008394) using the Matplotlib library are two examples of similar software that may be used to perform similar functions required to repeat this study.

## Results and discussion

### Proximate analysis

Table 1 shows the results of the proximate analysis for the samples. ^56^ Plukenetia volubilis seed on dry basis has a high lipid content (52.84%) like oilseeds such as soybean (16.61-24.71%), almond ( *Prunus dulcis*) and 72,620,13 peanut ( *Arachis hypogaea*) whose values vary around 50%. ^22^^,^^23^

**Table 1.** Proximate composition of flours.

Component	Sacha inchi almond	SIF-WM	SIF-DM	TF
% Protein	33.73 ^b^ ± 0.01	31.54 ^c^ ± 0.04	72.62 ^a^ ± 0.10	6.05 ^d^ ± 0.13
% Ashes	3.05 ^c^ ± 0.001	2.56 ^d^ ± 0.06	6.79 ^a^ ± 0.04	5.18 ^b^ ± 0.01
% Lipids	52.84 ^a^ ± 0.62	8.87 ^c^ ± 0.27	9.84 ^b^ ± 0.24	0.70 ^d^ ± 0.05
% Crude fiber	3.71 ^c^ ± 0.18	19.42 ^a^ ± 0.32	7.71 ^b^ ± 0.01	2.65 ^d^ ± 0.16
% Carbohydrates	6.67	37.61	3.04	85.42

The acronym (TF) refers to taro flour; (SIF-WM) sacha inchi flour by wet method and (SIF-DM) sacha inchi flour by defatted method. Values are presented as mean ± SD. For each parameter, different letters indicate significant differences at p < 0.05.

The results of lipids are within the range of values reported by Refs. 24– 27. The protein content of sacha inchi kernel (33.73%) is above the range, but close to the value reported by Ref. 25. As for ash, the value was 3.05%, crude fiber (3.71%), and carbohydrates (6.67%). Regarding moisture, sacha inchi seed presented a 6.62%, close to the value reported by Ref. 27 with 6.28%.

Two methods were used to obtain sacha inchi flour: the wet method and mechanical defatting.

Wet method: The flour obtained by the wet method had an oil content of 8.87% and a protein content of 31.54%, with a protein/fat ratio of 3.56.

Mechanical defatting method: the sacha inchi flour obtained by the mechanical defatting method had a protein content of 72.62%, being above the range of values reported by Refs. 24, 28– 31 which vary between 41.49 and 65.6%.

Crude fiber has a value of 7.71%, the carbohydrate content is 3.04%, below that reported by Ref. 28 with a value of 11.25%. This difference can result from suspended solids carried in the oil stream.

The comparison of the two methods for obtaining Sacha inchi flour (SIF-DM and SIF-WM) made it possible to see the method that presented the highest protein content was SIF-DM with 72.62% compared to a value of 31.55% for SIF-WM. The lipids results were close, 9.84% for the SIF-DM and 8.87% for SIF-WM. As for fiber, SIF-WM presented higher values (19.42%) compared to SIF-DM (7.71%).

Taro flour: as shown in Table 1, carbohydrates are the major component of taro with a value of 85.42%. The values of protein (6.05%), ash (5.18%), and lipids (0.70%) from proximate analysis agree with research. ^10^^,^^32^^–^^36^ Crude fiber is the only data below the range consulted with a value of 2.65%, the closest value being that reported by Ref. 36 with a value of 4.38%.

### Effect of processing on FPP and the antioxidant activities

Table 2 shows that the total polyphenols obtained in the flours are statistically different, taro flour presents the highest polyphenol content of 7.50 ± 1.73 mg EAG/ g, being like those reported by Ref. 37, in comparison with flours from other sources such as plantain (5.93 ± 0.43 mg EAG/ g) ^38^ present a higher value. The defatted sacha inchi flour presented a polyphenol content of 3.37 ± 0.16 mg EAG/g flour, being higher than the flour obtained by the wet method, because the mechanical extraction involving pressure and temperature allows a release of phenolic compounds. ^39^

**Table 2.** Bioactive properties of papachina and sachainchi flour.

Raw material	FPP (mg EAG/g sample)	ABTS (μmol ET/g sample)	a _w_
TF	7.47 ^a^ ± 0.27	2.71 ^a^ ± 0.02	0.61
SIF-WM	2.68 ^c^ ± 0.26	0.49 ^b^ ± 0.02	0.64
SIF-DM	3.37 ^b^ ± 0.16	0.71 ^c^ ± 0.04	0.54

The acronym (TF) refers to taro flour; (SIF-WM) sacha inchi flour by wet method and (SIF-DM) sacha inchi flour by defatted method; (FPP) refers free polyphenols. Values are presented as mean ± SD. For each parameter, different letters indicate significant differences at p < 0.05.

Regarding the antioxidant activity measured by the ABTS method, taro flour obtained a statistically higher antioxidant activity with 2.71 ± 0.02 μmol ET/g compared to SIF-DM (0.49 ± 0.02 μmol ET/ g) and SIF-WM (0.71 ± 0.04 μmol ET/g). This difference can be explained because taro is a prime source of antioxidant compounds and, from a chemical point of view, has various biologically active phytoconstituents, such as flavonoids, sterols, and glycosides. ^40^

In this study, a TF drying temperature of 60°C was used, which according to Ref. 41, temperatures between 50°C and 60°C allow for maximum antioxidant activities in taro flours. SIF-DM presented the lowest antioxidant activity because during extraction the oil stream carries away bioactive compounds, which in sacha inchi are present in essential fatty acids. ^42^

### Rheological analysis

*Pasting curves*

**Figure 1.**
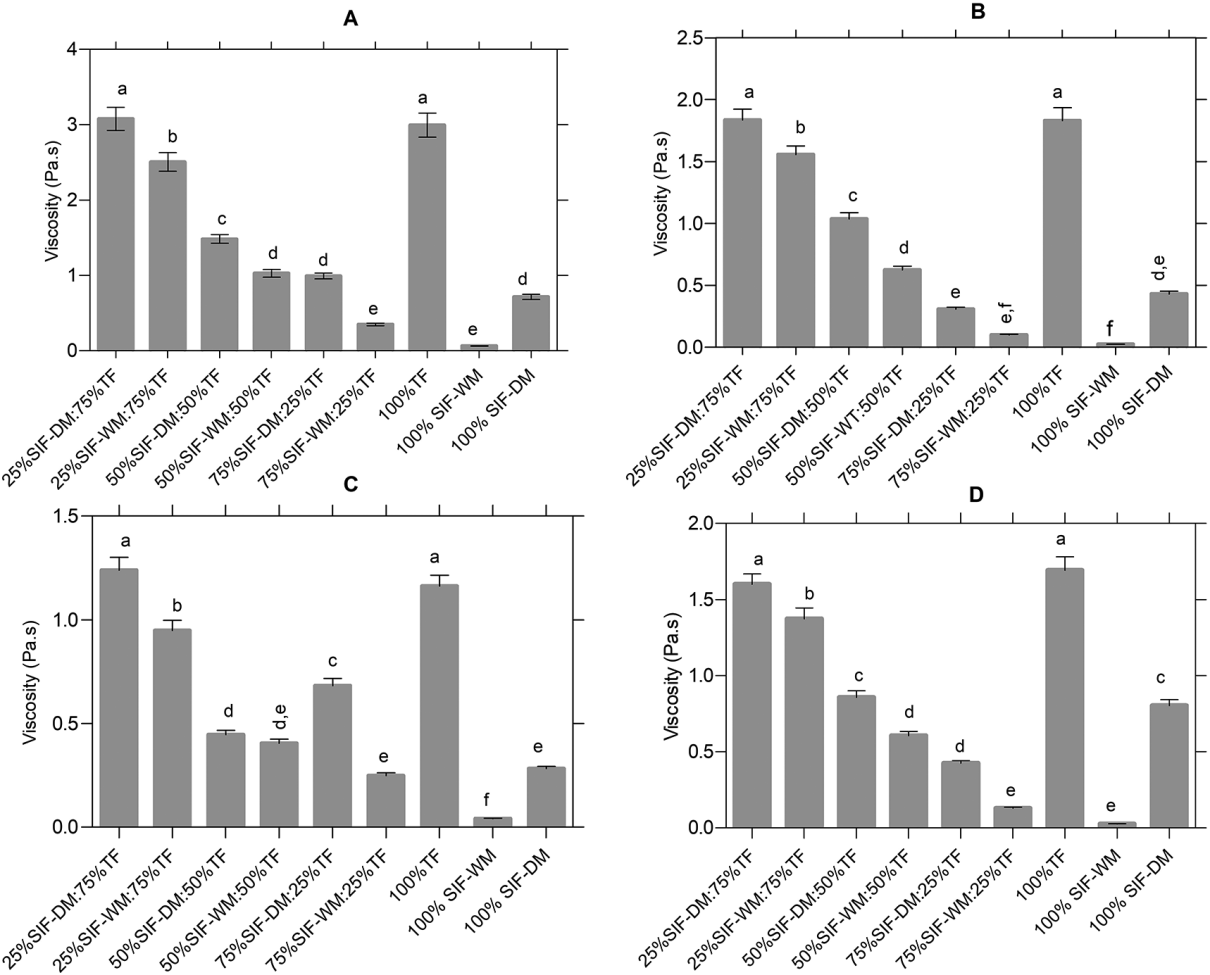
Effect of the different blends on the curves of the pasting curves. The data represent the significant differences in the values of: (A) viscosity peak [Pa.s]; (B) Trough [Pa.s]; (C) Breackdown [°C] and (D) setback [Pa.s]. The acronym (TF) refers to taro flour; (SIF-WM) sacha inchi flour by wet method and (SIF-DM) sacha inchi flour by defatted method. Values are presented as mean ± SD. For each parameter, different letters indicate significant differences at p < 0.05.

The viscosity profile was carried out to find the evolution of viscosity during the heating and cooling phase in the raw materials and each of the blends. According to the statistical analysis, the different flour blends significantly affect viscosity p < 0.05. Figure 1A shows the significant differences between the blends in the viscosity peak, where the presence of five different groups identified with letters from “a” to “e” are observed. In group “a”, the samples with the highest viscosity (25% SIF-DM:75% TF and 100% TF), and in group “e”, the samples with the lowest viscosity value (75% SIF-WM: 25% TF and 100% SIF-WM).

According to the results, the samples with higher TF content present the highest viscosity peaks, due to the presence of starch contributed by TF, taking into account that in this study taro, presented 85.42% of carbohydrates that according to ^43^ are represented by starch. Starch granules are not soluble in cold water, because of the strong hydrogen bonds that hold the starch chains together. Starch heated in excess water and above the pasting temperature suffers an order-disorder phase transition called gelatinization. This transition is associated with water diffusion into the granule, water uptake by the amorphous region, hydration, and radial swelling of the starch granules, resulting in an increase in viscosity. This positive correlation of starch content with peak viscosity is consistent with research on different commercial cereal, pseudo-cereal, root, and legume flours. ^44^ Comparing SIF-DM flour with SIF-WM, the defatting method produces flour with higher viscosity, because of the interaction of water with proteins and starch, ^19^ considering that SIF-DM has 72.62% protein.

Figure 1B shows the significant differences in the samples behavior before the onset of retrogradation (trough or drop viscosity), which occurs at the end of the constant temperature section, before cooling begins. There are six different groups, identified with letters from “a” to “f”, where in group “a”, we have the samples with the highest trough value (25% SIF-DM: 75% TF and 100% TF), and in group “f”, the samples with the lowest trough value (75% SIF-WM: 25% TF and 100% SIF-WM).

Accordingly, the samples that presented a greater viscosity drop are the ones with higher TF content, because of the alteration of the crystalline structure ( *amylopectin*) that makes up the granules, causing an increase in the size of the granules and partial solubilization of the starch. It is possible that in mixtures where the presence of starch provided by taro is in greater proportion and the protein provided by the sacha inchi result in more free water for the starch molecules to swell and solubilize; however, at high protein ratios, the association between protein and starch granules could play a more important role, stabilizing the amylose leaching channel of starch granules and resulting in lower swelling power and solubility. ^45^

Figure 1C shows the significant differences in the samples behavior in the breakdown stage. According to Ref. 46, the breakdown or stability is the difference between the viscosity peak and the trough in the constant temperature section and shows disintegration at a maintenance temperature (95°C) during continuous shear; the lower the Breackdown value, the higher the anti-shear capacity. In this study, we observed six different groups, identified by letters from “a” to “f”. In group “a”, the samples with the highest breakdown value (25% SIF-DM: 75% TF and 100% TF), and in group “f”, the samples with the lowest value (100% SIF-WM). It was evidenced that the samples with low starch content contributed by taro present higher stability, while the samples with higher starch content have lower capacity to support viscosity during prolonged heating, because of the starch behavior, in which the low molecular weight amylose separates from the starch granule and they collapse at constant temperature until the amorphous part is solubilized, decreasing viscosity. ^47^

Figure 1D shows the significant differences between the mixtures in the setback parameter, where five different groups are identified by letters from “a” to “e”. In group “a”, we find the samples with the highest setback (25% SIF-DM: 75% TF and 100% TF), and in group “e”, the samples with the lowest values (75% SIF-WM: 25% TF and 100% SIF-WM). The variable reorganization or setback is the indicator of the retrogradation and rearrangement of starch molecules during the cooling process that defines the reabsorption of soluble starch polymers and insoluble granular fragments during the cooling phase. ^48^ According to the results, blends containing lower taro content are less prone to retrogradation. As mentioned above, starch (amylose-amylopectin) contributed by TF with variations in temperature generates changes, e.g., amylose forms double helix associations of 40 to 70 glucose units, since amylopectin crystallization occurs by reassociation of the outermost short branches, i.e., both amylose and amylopectin are capable of retrogradation, although the amylopectin seems to be responsible for long-term quality changes in foods. ^49^ In taro starch, amylopectin contents are higher than amylose 66 and 34 % respectively, ^50^ which is the reason for attributing higher retrogradation in flour blends with higher TF.

*Flow profile analysis*

**Figure 2.**
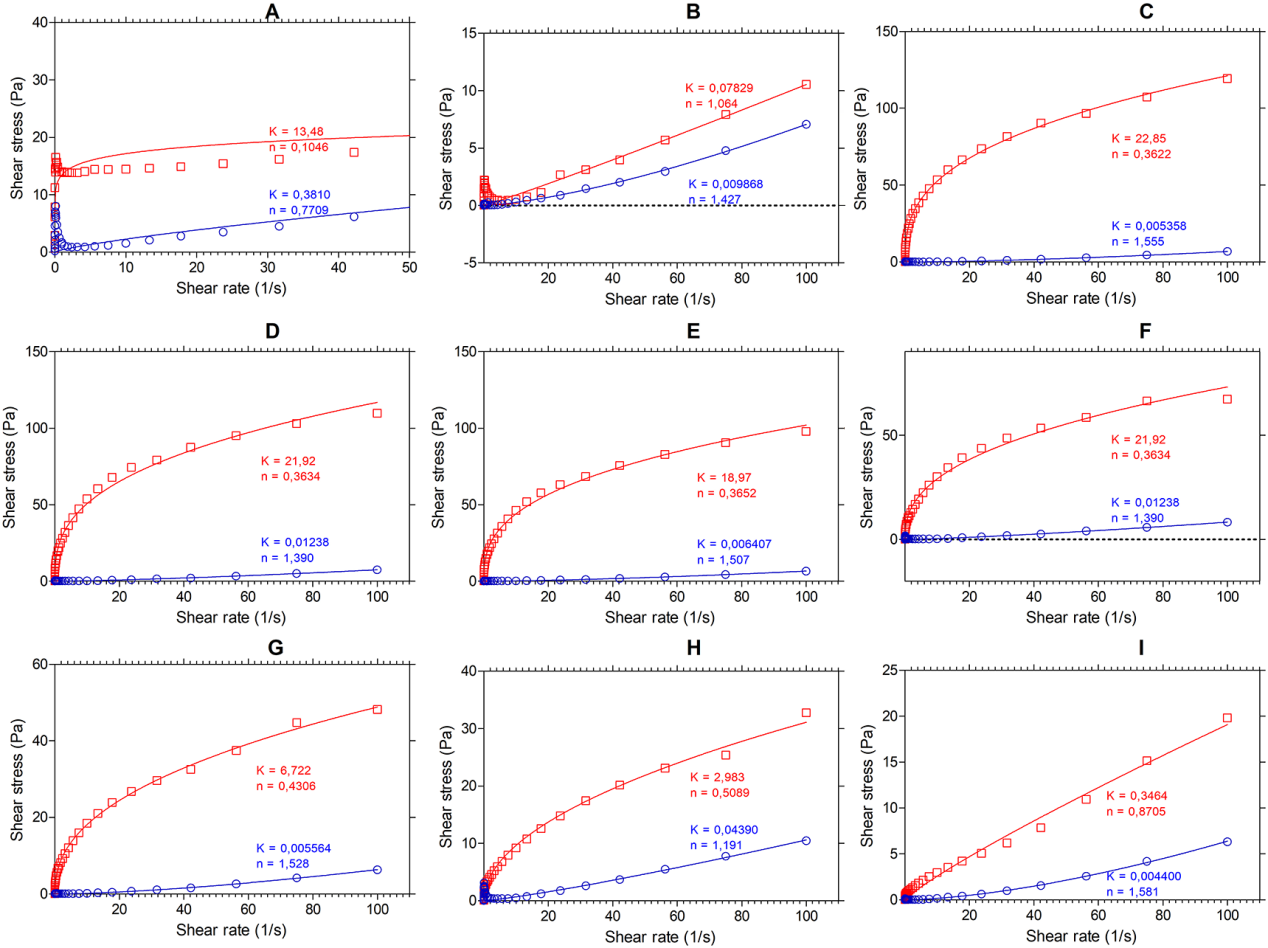
Flow profile of flour blends. The graphs show the flow profile behavior of: (A) SIF-DM; (B) SIF-WM; (C) TF; (D) 25% SIF-DM: 75% TF; (E) 25% SIF-WMH: 75% TF; (F) 50% SIF-DM: 50% TF; (G) 50% SIF-WM: 50% TF; (H) 75% SIF-DM: 25% TF; (I) 75% SIF-WM: 25% TF. The acronym (TF) refers to taro flour; (SIF-WM) sacha inchi flour by wet method and (SIF-DM) sacha inchi flour by defatted method.

Figure 2 shows the results of the flow analysis with the viscosity behavior before heating (blue line) and after heating (red line) under stress conditions (shear stress, Pa) and shear rate (s ^-1^). We observed the coefficients obtained by regression of the power model, where “n” is the flow index and “K” is the consistency index. According to the consistency index, we see that “K” increases in all samples except for SIF-WM, because of the viscosity gain, being “n” and “K” inversely proportional parameters.

Regarding the flowability index, we observed the behavior of the samples with the highest starch content TF were those that presented the greatest change in “n”, decreasing their flowability index represented by the gain in viscosity attributed to the behavior of the starch in taro.

Regarding the flow index, all the mixtures, except for SIF-DM, have a value of n > 1 before heating, showing that they have a dilettantish fluid behavior. In water and food starch suspensions, the dilatant or shear thickening behavior relates to the initial stiffness of starch granules to resist shear and to a high concentration of solids, resulting in particle swelling. ^51^ SIF-DM presented an n of 0.7709, demonstrating a behavior of a pseudo-plastic fluid.

For the mixture’s behavior after heating, all the blends, except for SIF-WM, presented an *n* < 1, demonstrating a behavior of a pseudo-plastic fluid, *i.e.,* that changes in temperature affect these mixtures behavior. This behavior occurs because, in the gelatinization process, the starch granules break, releasing amylose to the aqueous medium and, on cooling, these amylose chains align, forming networks that form gels or viscous suspensions. ^18^ The n values for the SIF-WM were reduced to 1.064, exhibiting Newtonian behavior. The Newtonian flow shows that the viscosity is independent of the shear rate. According to Ref. 51, these flours could be suitable for formulating products such as beverages, where improving the nutritional content is required without affecting its viscosity during shearing.

*Viscoelastic properties - Temperature sweeping*

**Figure 3.**
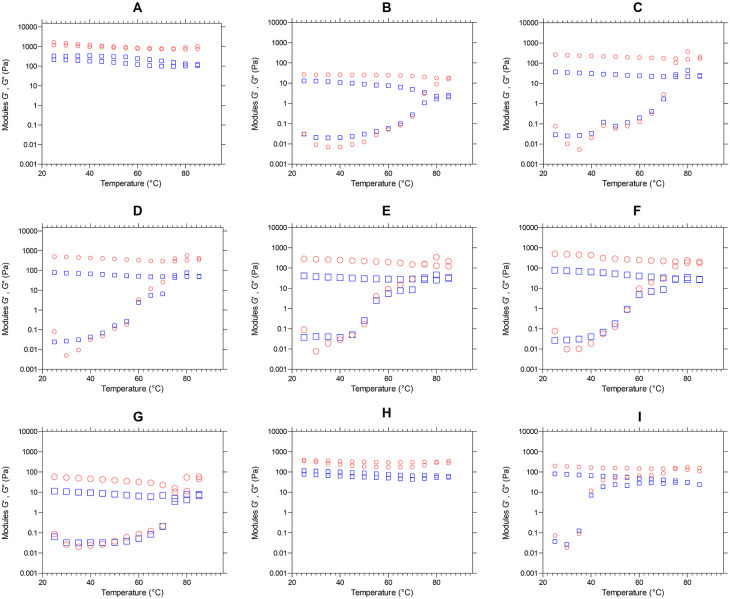
Temperature sweeping, modules G′ and G″ of the flour blends. The graphs show the loss modulus G″ and storage modulus G′ for: (A) SIF-DM; (B) SIF-WM; (C) TF; (D) 25% SIF-DM: 75% TF; (E) 25% SIF-WM: 75% TF; (F) 50% SIF-DM: 50% TF; (G) 50% SIF-WM: 50% TF; (H) 75% SIF-DM: 25% TF; (I) 75% SIF-WM: 25% TF. The acronym (TF) refers to taro flour; (SIF-WM) sacha inchi flour by wet method and (SIF-DM) sacha inchi flour by defatted method.

Figure 3 presents the moduli G′ (red color) and G″ (blue color) as a temperature function (°C). We observe the behavior of the elastic and viscous moduli for each mixture, evidencing an increase in the moduli as the temperature sweeps are performed. Except for graphs “A” and “H”, the mixtures behavior during heating is similar, since the two moduli increase their value, decreasing the difference between them and achieving a crosslinking, where the material ceases to have a viscous character, *i.e.,* it stops being liquid and becomes elastic properties characteristic of a solid. This behavior is characteristic of materials undergoing liquid-solid phase transformations. When the molecules gain weight, in this case, when the starch granules interlock, the loss modulus G" decreases and the storage modulus G′ increases, reaching a point of equilibrium that is its material change of nature, its solidification.

There is a progressive exudation of amylose in the swollen granules, where gel-like linkages are formed. Researchers have reported the effect of amylose to increase the firmness of gels during cooling as one of the initial causes of gel firmness. Thus, during the cooling stage, the moduli stay constant with the predominance of the elastic modulus. ^52^

The behavior of flour blends A and H corresponding to 100% SIF-DM and 75% SIF-DM: 25% TF, respectively, shows that the G′ and G″ moduli remain constant both during the temperature increase and during subsequent cooling, thus presenting greater stability to temperature variations. This behavior responds to the low starch content of these samples, since starch is key for the viscoelastic behavior of the flours, due to the interaction with water, generating absorption and swelling of the granule and, consequently, greater viscoelasticity. SIF-DM and 75% SIF-DM: 25% TF blends did not present a gel point, however, the other blends presented a gel-like behavior, highlighting that the 50% SIF-DM: 50% TF and 25% SIF-DM: 50% TF blends presented the most elastic gel at a gel temperature of 60°C in both cases, showing similar viscoelastic characteristics.

We suggest an optimum level of protein inclusion represented by sacha inchi between 25 and 50%, where the consistency of the gel increases and mixtures prepared above this range do not present a gel point, while lower values will have a more liquid behavior.

### Microbiological analysis

Table 3 shows the results of the microbiological analysis performed on the three types of flour. For the microbiological analysis, we considered the NTC 6069 ^53^ standard for quinoa flours and NTC 267 ^54^ for wheat flour.

**Table 3.** Microbiological analysis.

Microorganism	Specification (log UFC/g)	Counts (log UFC/g)		
		TF	SIF-DM	SIF-WM
Total mesophilic aerobic count	5.47	4.21 ± 0.09	4.32 ± 0.09	3.94 ± 0.05
*Staphylococos* count	<2	2.38 ± 0.09	1.52 ± 0.10	2.44 ± 0.08
Molds and yeasts count	3.7	1.51 ± 0.10	1.60 ± 0.08	2.02 ± 0.06
Total coliform count	<1	Absence	Absence	Absence
*Salmonella* in 25 g	Absence	Presence	Absence	Absence

The acronym (TF) refers to taro flour; (SIF-WM) sacha inchi flour by wet method and (SIF-DM) sacha inchi flour by defatted method.

As can be seen in Table 3, the flours comply with the quality specifications required by Colombian regulations; however, *Salmonella* is present in the taro flour, possibly due to the persistence of this type of microorganism; drying this flour at 60°C preserves some bioactive compounds but is not sufficient to eliminate it. According to Ref. 14, this type of pathogens acquire resistance to disinfectants and temperature because they form biofilms, which protect them and can remain in equipment, utensils and facilities, contaminating food. In addition, flours such as taro can be contaminated during harvesting or transportation.

Table 2 shows that in foods such as sacha inchi and taro flour, where a _w_ is less than 0.85, *Salmonella* and other pathogenic bacteria such as *Staphylococo* can survive in a viable but non-culturable state for long periods of time due to their increased resistance to thermal processes. ^55^ Although the growth of microorganisms is not maintained at low water activity, foodborne bacteria and fungi can easily contaminate flour and survive for long periods of time with a low reproduction rate. ^21^

## Conclusions

The method that allows for the highest concentration of protein in sacha inchi is mechanical defatting with a protein result of 73%, compared to that obtained by the wet method, which resulted in 31%. However, during the production of sacha flour with the mechanical method, bioactive compounds are lost, which is why lower values of total polyphenols and antioxidant activity are obtained, compared to taro flour.

The viscosity profile evaluation determined that the starch contained in taro provides good viscosity characteristics, being this an important ingredient for the development of foods such as creams, pastes, sauces, among others. However, mixtures with excess taro have a higher rate of retrogradation, presenting a disadvantage for food stability during storage.

Through the viscoelasticity analysis (temperature sweep), we found the optimum level of inclusion of defatted sacha inchi is between 25 and 50%, where a stable and consistent gel is presented.

Although taro’s drying temperature of 60°C maintains polyphenols and antioxidant activity, it does not allow the inactivation of bacteria such as *Salmonella*, which has shown resistance to thermal treatment in recent years.

Developing foods using gluten-free orphan raw materials, especially taro and sacha inchi that are easily adapted to regions with food safety hazards, can be a good alternative to offer the market different gluten-free products, excellent source of protein, and high fiber content.

Future research can formulate foods from sacha inchi flour, which will allow obtaining high protein foods, responding to the trend of obtaining high protein foods from vegetable sources.

*Salmonella* contamination was present in the taro flour. In response, the facilities and equipment were washed and disinfected with a different product than usual, and again salmonella analyses were performed to guarantee its elimination.

## Data Availability

Zenodo: Sacha_article, https://doi.org/10.5281/zenodo.7625871. ^56^ This project contains the following underlying data:
- REOLOGY SAMPLES.zip
• Flow profile analysis_folder containing data on the flow analysis of flours in different samples. (Folder contains data on flow analysis and statistical analysis of flours in different blends) • Pasting_containing curves and statistical data of pasting properties in different samples. (Folder containing curves and statistical data of pasting properties) • Viscoelasticity_containing curve data and statistical analysis of analyzed viscoelastic properties in different samples. (The folder contains the data of the curves and the statistical analysis of the viscoelastic properties analyzed) Zenodo: Sacha_article, https://doi.org/10.5281/zenodo.7625871. ^56^ This project contains the following extended data: Protocols on proximate and microbiological analysis
- Bacillus cereus--protocol for the determination of Bacillus cereus present in food according to the Colombian regulations is presented.docx. (This file contains the microbiology protocol for the determination of *Bacillus cereus* according to Colombian regulations) - Coliforms--protocol for the determination of coliforms present in food according to the Colombian regulations is presented.docx. (This file contains the microbiology protocol for the determination of Coliforms according to Colombian regulations) - Fungi and yeasts--protocol for the determination of fungi and yest present in food according to the Colombian regulations is presented.docx. (This file contains the microbiology protocol for the determination of Fungi and yeasts according to Colombian regulations) - Mesophiles-- protocol for the determination of mesophiles present in food according to the colombian regulations is presented.docx. (This file contains the microbiology protocol for the determination of Mesophiles according to Colombian regulations) - Salmonella Spp-- protocol for the determination of Samonella present in food according to the Colombian regulations is presented.docx. (This file contains the microbiology protocol for the determination of *Salmonella Spp* according to Colombian regulations) - Staphylococcus aureus--protocol for the determination of Salmonella present in food according to the Colombian regulations is presented.docx. (This file contains the microbiology protocol for the determination of *Staphylococcus aureus* according to Colombian regulations) - Protocol Fiber--protocol for fiber determination is presented.docx. (This file contains the proximal analysis protocol for fiber determination and handling of the equipment used for this determination) - Protocol Lipid--protocol for lipds determination is presented.docx. (This file contains the proximal analysis protocol for lipds determination and handling of the equipment used for this determination) - Protocol Protein--protocol for protein determination is presented.docx. (This file contains the proximal analysis protocol for protein determination and handling of the equipment used for this determination.) Data are available under the terms of the Creative Commons Attribution 4.0 International license (CC-BY 4.0).
