# Characterization of sacha inchi (Plukenetia volubilis) and taro (Colocasia esculenta) flours with potential application in the preparation of both gluten-free and high protein foods.

**DOI:** 10.12688/f1000research.130394.4

**Published:** 2025-03-07

**Authors:** Ruby Aracely Narváez Cadena, Angie Paola Salas Zambrano, Jesús Eduardo Bravo Gómez, Karen Sofia Muñoz Pabon, Diego Fernando Roa-Acosta

**Affiliations:** 1 Departamento de Agroindustria, Universidad del Cauca, Popayan, Colombia

**Keywords:** Rheology properties, Proximal composition, Microbiological quality, Gluten-free foods

## Abstract

**Background:**

Interest in alternative sources of tubers and legumes has increased due to the search for raw materials with bioactive antioxidant compounds. The objective of this study was to characterize taro (TF) and sacha inchi ( *Plukenetia volubilis*) flours obtained by the wet (SIF-WM) and defatted (SIF-DM) methods, as alternatives for the formulation of functional foods.

**Methods:**

Proximal tests were conducted to determine basic chemical composition, quantification of free polyphenols, antioxidant activity using the ABTS* radical method (2,2′-azino-bis(3-ethylbenzothiazoline-6-sulfonic acid)) with Trolox as a standard, and rheological analyses, including pasting curves, flow profiles, and viscoelastic properties. Microbiological characterization of the flours was also performed.

**Results:**

The highest protein content was found in sacha inchi flour obtained by the defatted method (72.62%), while carbohydrates were the main component in taro flour (85.4%). In terms of antioxidant activity, taro flour showed higher values of 2.71 µmol ET/g and 7.47 mg EAG/g. Rheological analysis showed that adding taro flour increased the viscosity peak and reduced breakdown, improving gel stability. *Staphylococcus aureus* and *Salmonella spp.* were detected in taro flour.

**Conclusions:**

Taro and sacha inchi flours have great potential for the development of functional products like protein snacks, with good expansion due to taro starch and are naturally gluten-free, making them suitable for people with celiac disease.

## Introduction

According to Wang et al., ^1^ by 2050, if global governance fosters debate and ensures equity in the food system, and if food demand increases significantly due to rapid population growth, intensive land use will be required to meet the demand, particularly for meat and dairy products, which are vital sources of protein, vitamins, and minerals in the human diet. ^2^ However, intensive livestock production is environmentally unsustainable due to its high demand for resources, such as land for growing animal feed and fresh water. Furthermore, this activity significantly contributes to greenhouse gas emissions, exacerbating global warming. ^3^ Consequently, a viable strategy to ensure adequate protein intake is to adopt an optimal combination of plant-based proteins.

In this scenario, we must design food systems to contribute to global environmental sustainability and meet nutritional needs, proposing diets based on plants that allow including adjustments according to each region coupled with cultural customs. ^4^ Underutilized or orphan crops are used to design original foods, not aimed at international trade, however, because of their high adaptation to the local environment, these crops play an important role in regional nutritional security. ^5^ The mixture of plants such as cereals, pseudocereals, legumes, tubers, among others, are used to create different products with adequate balance of protein, carbohydrates, fiber and micronutrients such as vitamins and minerals.

For example, sacha inchi ( *Plukenetia volubilis*) known as wild peanut, Inca peanut, sacha peanut or mountain peanut, is an oleaginous plant that belongs to the Euphorbiaceae family. ^6^ Today, sacha inchi is still cultivated in the lowlands of the Peruvian Amazon and has been planted for centuries by the indigenous population. ^7^ In Colombia, production exceeds 2,400 tons of sacha inchi seed; the department of Putumayo is the largest producer of sacha inchi with 282 hectares, followed by Valle del Cauca, Caquetá and Antioquia. ^8^

Sacha inchi is a promising and industrializable food native to the Amazon that has essential unsaturated fatty acids such as omega-3, as alpha-linoleic acid with 47.7% to 51.9% and omega-9 as oleic acid with 7.9 to 8.9% by weight of oil, 27.4% protein, 4% ash and about 50% oil. ^9^ These nutritional properties make sacha a favorable food for health and a suitable crop for developing high-protein and gluten-free foods. However, sacha inchi can be unstable due to its high concentration of unsaturated fatty acids, which makes it sensitive to oxidation. ^10^ In addition, sacha inchi has limiting amino acids such as lysine and tryptophan that can cause low digestibility. ^11^ Likewise, this seed has anti-nutritional factors ^12^ that limit its use as raw material in the development of new products.

Taro is a tuber abundant in starch of which 17-28% is amylose and the remaining is amylopectin. ^13^ Taro has a high content of resistant starch that allows slow digestion with valuable effects on cholesterol and blood glucose regulation. ^14^ Taro has a high carbohydrate (59.36%) and protein (24.99%) content, with respect to the mineral content of taro, the highest amount present is magnesium 242.373 mg/kg, followed by calcium 94.455 mg/kg, iron 8.351 mg/kg and zinc 6.210 mg/kg, and, in relation to vitamins, is reported content of vitamin C 0.188 mg/100 mg, vitamin B1 0.047 mg/100 mg, and vitamin B3 0.078 mg/100 mg. ^15^ Compared to other tubers such as sweet potato, potato, cassava and yam; taro has a higher protein and fat content. ^15^ Likewise, taro has flavonoids and phenolic acids that have antioxidant properties; flavonoids, the largest group of phenolic compounds identified in the whole plant, are associated with reducing many degenerative diseases. ^14^ However, taro contains antinutrients such as oxalates, which can limit its consumption in fresh form due to the irritation they cause in the throat and mouth. ^16^ Nevertheless, the extrusion process significantly reduces these drawbacks and simultaneously improves nutritional properties, such as increased protein digestibility. Based on this, combining Sacha inchi and taro is an excellent option to complement each other’s nutritional properties, enhancing nutritional value and improving the stability and texture of the final product.

Foodstuffs are complex systems of great nutritional richness and therefore sensitive to attack by microorganisms (bacteria, fungi and yeasts). The main mode of contamination of raw materials is animal defecation, manure fertilization and recontamination persisting in facilities and transport; in addition, insects and rodents are a source of contamination. ^17^ The low water activity (a _w_<0.60) of meal does not favor microbial growth; however, contaminating spores along with inactive microorganisms will remain viable for prolonged periods and constitute a potential health hazard. As a persistence mechanism, *Salmonella spp.* and other pathogens form biofilms that protect against disinfection and increase their tolerance to drying processes. ^17^

In food matrices there is always a microbial load that must not exceed certain limits, according to Colombian regulations, which must be controlled to avoid the deterioration of the product and the consequent loss of its quality and suitability for consumption.

The design and production of new foods that respond to food and nutritional security in the midst of climate change in low-income countries is a challenge, so this study aimed to characterize the chemical, microbiological, rheological and bioactive properties of individual flours and mixtures of taro and sacha inchi, as potential foods applied in the formulation of different foods.

## Methods

The taro tubers were bought in the Municipality of Orito Putumayo, located at 0° 38′ North Latitude and 76° 37′ West Latitude of Greenwich. Average temperature of 25 °C and relative humidity of 88%. The fresh sacha inchi almonds were supplied by the company Fruty Amazónicos S.A.S. located in the village of La Concordia in the municipality of Valle del Guamuez, located at 00° 25″ north latitude and 76° 54″ west longitude. Temperatures range between 27 °C and 40 °C.

### Obtaining taro flour

Taro flour (TF) was obtained following the methodology described by Quezada Correa et al. ^13^ The raw material was received, the peel was removed; it was washed to eliminate impurities and then the edible part was cut into slices to facilitate dehydration; then it was weighed on a FENIX Electronic Weight Only Table Scale to verify yield, subsequently it was dried in a rotary oven (ORVES, Colombia) at a temperature of 60 °C for five hours to eliminate excess moisture. The dried slices were subsequently ground in an electric mill (Quaker City Mill, model 4-E, Philadelphia) and then sieved through a 30-mesh system to achieve homogeneity in the flour particle size. They were then stored in airtight bags until further use.

### Obtaining sacha inchi flour

The sacha inchi seeds were received as white kernels, meaning without shell or husk. For the compositional analysis of the kernel, they were manually ground with a mortar to reduce their size, thus facilitating the relevant tests.

*Wet method*

The sacha inchi flour obtained by the wet method (SIF-WM) was prepared as follows: initially, wet grinding was carried out using a blender (Oster, BLST 4655, Colombia) with a capacity of 1.25 L. For this process, sacha inchi seeds were mixed with water in a 1:3 ratio to ensure effective grinding. This resulted in a milky and homogeneous suspension. Subsequently, a cloth filter was used to separate the insoluble extract (cake) from the water-soluble extract (milk). The cake was then dried in an oven at 65 °C for 3 hours. Finally, once dehydrated, it was immediately packaged in a polyethylene bag with a hermetic seal for preservation. ^18^

*Defatted method*

**Figure 1.**
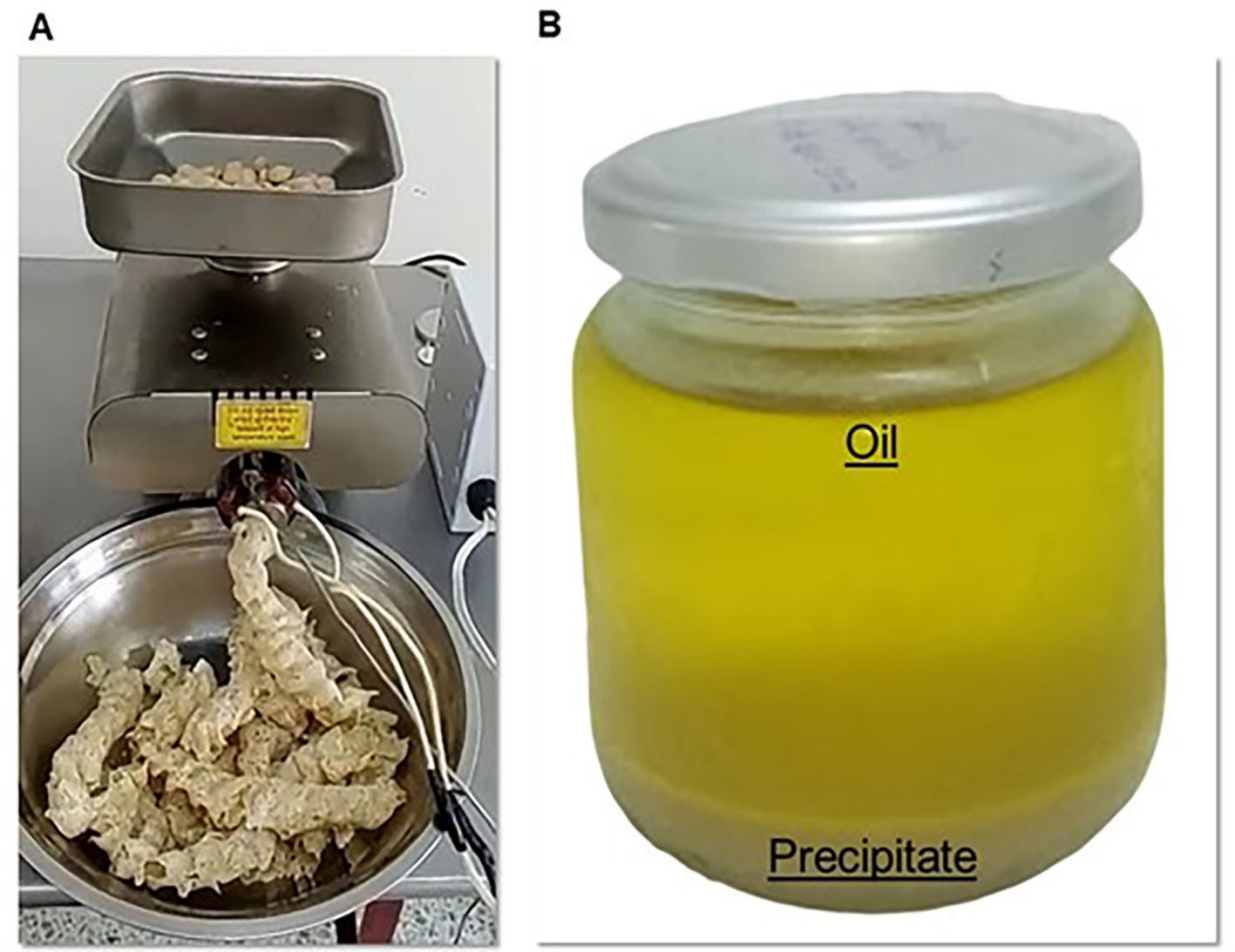
(A) Defatting process of sacha inchi, (B) Oil obtained after mechanical extraction.

The defatted sacha inchi flour (SIF-DM) was obtained as follows: the sacha inchi kernels were placed in the hopper of an automatic touchscreen oil extraction press (CGLDENWALL, model K28, Shanghai, China), as shown in Figure 1. The process was carried out at a temperature of 124 °C. During the extraction, a screw conveyor pushed the material into the main pressing cylinder. As it moved forward, the design of the screw, with decreasing pitch and spiral depth, reduced the available volume in the chamber, subjecting the material to high pressure and friction against the closed bottom wall of the cylinder. This facilitated efficient oil extraction, with the oil flowing through the cylinder’s orifices, while the residual material, known as cake, was expelled through the discharge nozzle. Subsequently, the cake was ground using an electric mill (Quaker City Mill, model 4-E, Philadelphia) and sieved through a system with a No. 30 mesh to achieve uniform particle size. Finally, the resulting flour was packaged in polyethylene bags with airtight seals for preservation.

### Proximate analysis

The proximate composition (protein, lipids, dietary fiber, ash and moisture) of taro and sacha inchi flours was determined according to the methods proposed by the AOAC (Association of Official Analytical Chemists, 1990) ^19^ and the carbohydrate content was estimated by difference. The details of the method are explained in more detail in the protocols uploaded in the repository.

*Water activity measurement*

To determine the a _w_ of the raw materials, the powdered sample was placed in a Decagon Pawkit portable water activity meter until the cuvette was covered. Before reporting the measurement, the equipment was calibrated with standard salts at 0.25 (13.41 mol/Kg LiCl 0.25 a _w_) and 0.76 (6.00 mol/Kg NaCl 0.76 a _w_).

*Protein*

For the determination of protein content, the protocol established by AOAC 968.06 was followed. On nitrogen-free paper, 0.2 to 0.8 g of sample plus 1 g of kjeldahl catalyst were weighed and placed in digestion tubes. 10 mL of sulfuric acid were added. Then, gentle heating was initiated until no foaming or splashing was observed, using a temperature ramp as follows: 125 °C for 30 min, 270 °C for 30 min and 400 °C for 140 min.

The samples were digested until they were completely clear and translucent, free of organic matter, in the laboratory Kjeldahl digester (Raypa MBC-6/N, Spain).

Then they were cooled to room temperature in the Raypa distillation unit, after which each sample was titrated with 0.1 N HCl.

*Lipids*

For lipid testing, 1 g of sample was weighed in the extraction cartridges, then 80 mL of petroleum ether was added, immediately transferred in the rack to the laboratory Soxhlet and Randall Extractor (SX-6MP, RAYPA, Spain). After the extraction time and solvent recovery, the samples were placed in an oven at 60 °C for 1 hour to eliminate the remaining ether.

*Fiber*

The fiber content was analyzed according to the AOAC 962.09 protocol. For fiber testing, 1-2 g of the degreased sample were transferred to the laboratory fiber extractor (F-6P Fibertest, Spain) and fixed to the angle of the front part of the unit. Then 150 mL of 0.255 N H _2_SO _4_ was heated in an Erlenmeyer flask, when it was boiling, it was placed on top of the coolant, the heating knob was adjusted to boiling point 3 or 4 and left boiling for 30 minutes. At the end of this time, it was filtered and washed with distilled water and the operation was repeated 3 times using 30 mL of water each time. Then, 150 mL of sodium hydroxide solution preheated to 90 °C was placed in the upper part of the cooler; it was brought to boiling and kept for another 30 minutes. Then it was filtered and washed three times with boiling water. The sample was then placed in an oven at a temperature of 100-110 °C until a constant weight was obtained.

### Ash

The ash content was determined following the protocol established by AOAC 942.05. Empty porcelain crucibles were dried in an oven at 100 °C for 3 hours and then transferred to a desiccator to cool. Each crucible was weighed, and the value was recorded. Subsequently, 1 gram of dry sample was weighed into each porcelain crucible and ashed at 500-550 °C for 3 hours in a muffle furnace. After this time, the crucibles were transferred to the desiccator, allowed to cool, and then weighed.

### Determination of free polyphenols, FPP fraction

The determination of polyphenols was performed according to Muñoz Pabón et al., ^20^ with slight modifications. Two grams of the sample were weighed in a 50 mL falcon tube. The first fraction was added to the sample 8 mL of solution with 80% ethanol and 20% water and 1% formic acid (80 mL ethanol + 20 mL water + 1 mL formic acid). The samples were shaken for 25 minutes in a shaker at 200 RPM at room temperature. The samples were then centrifuged at 3500 rpm for 5 minutes at room temperature. Subsequently, the supernatant was taken in a new falcon tube (50 mL) and then 40 microliters of EDTA 2% was added to the supernatant.

For the second extraction, 8 mL of 70% acetone with 1% formic acid (70 mL acetone + 30 mL water + 1 mL formic acid) was added to the pellet from the first extraction. The pellet was shaken in the solution for 25 minutes in the shaker at 200 RPM at room temperature. Subsequently, the samples were centrifuged at 3500 RPM for 5 minutes at room temperature, and the second supernatant was combined with the first supernatant and shaken. The resulting mixture was made up to 20 mL using distilled water. Next, the measurement was carried out using the Folin-Ciocalteu method. 20 μL of the extract was taken and combined with 900 μL of Folin reagent, vortexed, and then 600 μL of NaHCO _3_ was added, shaking again to ensure proper dissolution. The reaction was allowed to rest, and finally, the absorbance was measured at 765 nm in the spectrophotometer (Thermo Scientific, Genesys 10S UV VIS).

### Determination of antioxidant activity by the ABTS method

In a test tube, 4 mL of ABTS.* solution (2,2′-azino-bis(3-ethylbenzothiazoline-6-sulfonic acid)) was added, followed by 135 μL of the standard solution, and it was vortexed for 5 seconds. For the reagent blank, a mixture of 4 mL of 4.5 acetate buffer and 135 μL of ethanol was used. The zero point was prepared with 4 mL of ABTS solution and 135 μL of ethanol. The tube was then capped and allowed to rest for exactly 30 minutes before measuring the absorbance in the spectrophotometer (Thermo Scientific, Genesys 10S UV VIS) at a wavelength of 729 nm.

### Rheological analysis

*Pasting properties*

The pasting properties of each dispersion were determined using a rheometer (TA INSTRUMENTS, AR 1500, New Castel, USA), equipped with a starch pasting cell. Then, a suspension of flour in water with a concentration of 12% (w/w) in 25 g was prepared and exposed to heating and cooling. The following samples were analyzed: 100%TF; 100%SIF-DM; 100%SIF-WM; 25%SIF-DM:75%TF; 50%SIF-DM:50%TF; 75%SIF-DM:25%TF; 25%SIF-WM:75%TF, 50%SIF-WM:50%TF, 75%SIF-WM:25%TF. The shear rate was kept constant at 16.75 s ^-1^, throughout the heating and cooling range (25 °C–90 °C–25 °C) while the heating rate was 10 °C/min. The following parameters were obtained from the rheological analysis maximum viscosity [Pa.s]; minimum viscosity [Pa.s]; breakthrough viscosity [°C] and setback viscosity [Pa.s]. Finally, using the Savistky-Golay function, the data were smoothed in the GraphPad Prism 8.0.1 program (RRID:SCR_002798). ^21^^,^^22^

*Flow profile*

The flow properties were determined following the methodology outlined by Polo and Roa, ^21^ with some modifications. An AR1500 rheometer (TA Instruments, New Castle, USA) was used for this study, and measurements were taken using cylindrical geometry. The average viscosity was determined at 25 °C for 12 minutes, with the shear rate increasing in 4 steps as follows:

Step 1: 1×10 ^-3^ s ^-1^ to 1×10 ^-2^ s ^-1^

Step 2: 0.01 s ^-1^ to 0.1 s ^-1^

Step 3: 0.1 s ^-1^ to 1 s ^-1^

Step 4: 1 s ^-1^ to 100 s ^-1^

Subsequently, the sample was heated to 90 °C. Once this process was completed, the average viscosity was determined again at 25 °C, subjecting the sample to the same shear rate.

Flow curves (shear stress versus shear rate) were obtained and fitted to the power law model shown in Equation 1 *.*

$$T=K*y^{n}$$ (1)

Where:

T is the shear stress (Pa).

*y* is the shear velocity (s ^-1^)

*K* is the coefficient of consistency (Pa. s ^-1^)

*n* is the flow behavior index

The flow behavior index indicates Newtonian flow behavior when *n* = 1, shear thinning behavior when *n* < 1 and shear thickening when *n* > 1). Consistency and creep were determined before and after heating in order to determine the effect of thermal processing on these parameters.

*Determination of viscoelastic properties (temperature sweep)*

For the determination of viscoelastic properties (temperature sweep), the method described by Roa et al. ^23^ was used. The viscoelastic properties were determined in a rheometer (TA INSTRUMENTS, AR 1500, New Castel, USA) using a system of parallel flat plates, of 40 mm diameter and 1500 μm distance between plates. The edges of the plates were sealed with vaseline, with the purpose of controlling evaporation and avoiding variations in concentrations of the aqueous suspensions used.

The samples were subjected to a cycle of dynamic heating (25-85 °C), stabilization (85 °C for 2 minutes) and cooling (85-25 °C) at 10°C/min, with a frequency of 0.5 Hz and 0.5% deformation. The profiles of the viscoelastic moduli as a function of temperature were recorded using the equipment’s software.

### Microbiological characterization

The microbiological characterization of the flours was carried out, following the methodology reported by Muñoz Pabon et al., ^24^ which was based on parameters established by the Colombian technical standard NTC that applies for each microorganism.

To perform the microbiological characterization, 10 g of sample were taken in triplicate, then diluted in 90 mL of peptonized water and mixed at 150 rpm for 10 min in a shaker (MaxQ 4450 orbital Thermo Ficher Scientific USA), following this, for each microorganism the seeding of the appropriate dilution and selective medium is described. The details of the method are explained in more detail in the protocols uploaded in the repository https://doi.org/10.5281/zenodo.7582202.

*Coliforms*

1 mL of each replicate was taken and seeded in a previously sterile Petri dish by seeding in depth on chromogenic colinstant agar for 24-48 h of incubation at 35 °C.

*Mesophiles*

1 mL of sample was taken from each replicate, and each dilution was seeded by immersion in previously sterile Petri dishes in Plate Count Agar (PCA) agar for 24-72 h at 30 °C.

*Fungi and yeasts*

100 μL of each replicate was taken and seeded per surface in previously sterile Petri dish by surface plate seeding on Potato Dextrose Agar (PDA) agar for 24-72 h incubation at 30 °C.

*Bacillus cereus*

100 μL of each replicate was taken and surface seeded in previously sterile Petri dish by surface plate seeding on Mannitol egg Yolk Polymyxin agar (MYP) agar with egg yolk specific for *Bacillus cereus* for 24 h incubation at 37 °C.

*Staphylococcus aureus*

100 μL were taken from each replicate and seeded per surface in a previously sterile Petri dish, by surface plate counting on Baird Parker agar at 24 h incubation at 35 °C, and then incubated for 24 h at 35 °C.

*Salmonella spp.*

25 g of sample were taken in duplicate, then diluted in 225 mL of buffered pepton water and mixed at 150 rpm for 10 min in shaker (MaxQ 4450 orbital Thermo Ficher Scientific USA), once this time was over, they were left in incubation at 37 °C for 18 h, after which 100 μL were inoculated in 10 mL of Rappaport Vassiliadis malachite green broth (RVS medium) at 41. Furthermore, 1000 μL were inoculated in 10 mL of Tetrationate Mueller Kauffmann broth (MKTTn medium) at 37 °C for 24 h. After this time, the surface of a Petri dish containing XLD agar selective medium was seeded by means of a loop in such a way that asylated colonies were obtained, Salmonella Shiguella agar was incubated at 34 °C for 24 h, at the end of which typical colonies of *Salmonella spp.* were observed.

### Statistical analysis

A completely randomized design (nine treatments) was used to evaluate the rheological properties of native and formulated flours. The design was based on the type of flour and its inclusion levels in the mix, which were: 100%TF; 100%SIF-DM; 100%SIF-WM; 25%SIF-DM:75% TF; 50%SIF-DM:50%TF; 75%SIF-DM:25%TF; 25%SIF-WM:75%TF, 50%SIF-WM:50%TF, 75%SIF-WM:25%TF. The response variables were viscosity as a function of temperature-shear rate and viscoelastic modulus (G′ and G″).

The results were presented as the mean ± standard deviation of triplicate experiments. One-way analysis of variance (ANOVA) was used to compare means. Differences between means were considered significant at P < 0.05 using Tukey’s new multiple range test. Data were subjected to analysis with Minitab version 20 (RRID:SCR_014483), https://www.minitab.com/es-mx/support/downloads/. Graphs were generated in Graphpad Prism 5.0.

## Results and discussion

### Proximate analysis

Table 1 shows the results of the proximate analysis for the samples. Plukenetia volubilis seed on dry basis has a high lipid content (52.84%) like oilseeds such as soybean (16.61-24.71%), almond ( *Prunus dulcis*) and peanut ( *Arachis hypogaea*) whose values vary around 50%. ^25^^,^^26^

**Table 1.** Proximate composition of flours.

Component	Sacha inchi almond	SIF-WM	SIF-DM	TF
% Protein	33.73 ^b^ ± 0.01	31.54 ^c^ ± 0.04	72.62 ^a^ ± 0.10	6.05 ^d^ ± 0.13
% Ashes	3.05 ^c^ ± 0.001	2.56 ^d^ ± 0.06	6.79 ^a^ ± 0.04	5.18 ^b^ ± 0.01
% Lipids	52.84 ^a^ ± 0.62	8.87 ^c^ ± 0.27	9.84 ^b^ ± 0.24	0.70 ^d^ ± 0.05
% Crude fiber	3.71 ^c^ ± 0.18	19.42 ^a^ ± 0.32	7.71 ^b^ ± 0.01	2.65 ^d^ ± 0.16
% Carbohydrates	6.67	37.61	3.04	85.42

The acronym (TF) refers to taro flour; (SIF-WM) sacha inchi flour by wet method and (SIF-DM) sacha inchi flour by defatted method. Values are presented as mean ± SD. For each parameter, different letters indicate significant differences at p < 0.05.

The lipid results are within the range of values reported by Ruiz et al., ^27^ Benítez et al., ^28^ Bueno-Borges et al., ^29^ and Arévalo et al. ^30^ The protein content of sacha inchi kernel (33.73%) is above this range but close to the value reported by Benítez et al. ^28^ Regarding ash, the value obtained was 3.05%, crude fiber was 3.71%, and carbohydrates accounted for 6.67%. In terms of moisture, sacha inchi seeds presented a content of 6.62%, which is close to the value reported by Arévalo et al., ^30^ who reported 6.28%.

Two methods were used to obtain sacha inchi flour: the wet method and mechanical defatting.

Wet method: The flour obtained by the wet method had an oil content of 8.87% and a protein content of 31.54%, with a protein/fat ratio of 3.56. The high protein values obtained from sacha inchi cake using this mechanical pressing method are the basis for the formulation of protein-rich foods using vegetable sources.

Mechanical defatting method: The sacha inchi flour obtained by the mechanical defatting method had a protein content of 72.62%, exceeding the range of values reported by Ruiz et al., ^27^ Benítez et al., ^28^ Bueno-Borges et al., ^29^ Arévalo et al., ^30^ and González-Linares et al., ^31^ which vary between 41.49% and 65.6%.

Crude fiber content was 7.71%, while carbohydrate content was 3.04%, which is lower than the 11.25% reported by González-Linares et al. ^31^ This difference may result from suspended solids carried in the oil stream.

The comparison of the two methods for obtaining sacha inchi flour (SIF-DM and SIF-WM) made it possible to see the method that presented the highest protein content was SIF-DM with 72.62% compared to a value of 31.55% for SIF-WM. The lipids results were close, 9.84% for the SIF-DM and 8.87% for SIF-WM. As for fiber, SIF-WM presented higher values (19.42%) compared to SIF-DM (7.71%).

It is important to note that the protein content obtained through the wet milling method is lower compared to the mechanical defatting method. This is because, during the wet milling process, a milky suspension is generated, which is then filtered to separate the insoluble extract (cake) from the water-soluble extract (slurry). During filtration, part of the protein may remain in the soluble extract, which explains the lower protein concentration in the cake. In this study, the proximate analysis was performed only on the insoluble extract. ^32^^,^^33^

Regarding carbohydrate content, SIF-DM showed a lower percentage compared to other extraction methods. This can be explained by the fact that during the mechanical oil extraction process, not only fat is removed but also some solids, such as proteins, minerals, and certain carbohydrates, which could become trapped in the oil or be eliminated along with the byproducts generated during extraction (see Figure 1B). On the other hand, it is likely that carbohydrates are solubilized in the water used in the wet method, contributing to their higher content in SIF-WM. Additionally, the higher carbohydrate content observed in SIF-WM could be due to a proportional effect: the reduction in proteins during the wet process increases the relative proportion of carbohydrates, which are calculated by difference. Moreover, the wet method may promote the retention of insoluble carbohydrates within the solid matrix, minimizing the loss of hydrophilic compounds and explaining the higher carbohydrate content in SIF-WM.

Taro flour: as shown in Table 1, carbohydrates are the major component of taro with a value of 85.42%. The values of protein (6.05%), ash (5.18%), and lipids (0.70%) from proximate analysis agree with research. ^13, 37– 41^ Crude fiber is the only parameter below the consulted range, with a value of 2.65%, the closest being the 4.38% reported by Calle et al. ^41^

The study design was based on the type of flour and its inclusion levels in the mixture, considering protein content and carbohydrate percentage as the most relevant factors in each treatment. The proximal composition of the blends was calculated using the individual results obtained from each flour. The analyses showed that the 25%SIF-DM:75%TF blend contained 22.69% protein and 64.83% carbohydrates; the 50%SIF-DM:50%TF blend showed 39.34% protein and 44.23% carbohydrates; and the 75%SIF-DM:25%TF blend reached 55.98% protein and 23.64% carbohydrates. On the other hand, the blends with SIF-WM presented the following values: 25%SIF-WM:75%TF with 12.42% protein and 73.47% carbohydrates; 50%SIF-WM:50%TF with 18.80% protein and 61.52% carbohydrates; and finally, the 75%SIF-WM:25%TF blend with 25.17% protein and 49.56% carbohydrates.

### Effect of processing on FPP and the antioxidant activities

Table 2 highlights statistically significant differences in the total polyphenol content among the flours analyzed. Taro flour exhibited the highest polyphenol content, aligning with findings reported by Eleazu et al. ^42^ and surpassing other food sources such as plantain flour, as noted by. ^43^ Meanwhile, defatted sacha inchi flour demonstrated a higher polyphenol content compared to the flour obtained through the wet method. This result is likely due to the mechanical extraction process, which involves pressure and temperature, facilitating the release of phenolic compounds, as previously observed. ^44^

**Table 2.** Bioactive properties of papachina and sachainchi flour.

Raw material	FPP (mg EAG/g sample)	ABTS (μmol ET/g sample)	a _w_
TF	7.47 ^a^ ± 0.27	2.71 ^a^ ± 0.02	0.61
SIF-WM	2.68 ^c^ ± 0.26	0.49 ^c^ ± 0.02	0.64
SIF-DM	3.37 ^b^ ± 0.16	0.71 ^b^ ± 0.04	0.54

The acronym (TF) refers to taro flour; (SIF-WM) sacha inchi flour by wet method and (SIF-DM) sacha inchi flour by defatted method; (FPP) refers free polyphenols. Values are presented as mean ± SD. For each parameter, different letters indicate significant differences at p < 0.05.

Antioxidants present in foods play an important role in their preservation because they prevent oxidation processes; in addition, these compounds contribute to health promotion through the prevention of pathological processes mediated by oxidative stress. Therefore, the formulation of new foods that present bioactive compounds is attractive for the new trends in the design of new foods.

Regarding the antioxidant activity measured using the ABTS method, taro flour demonstrated significantly higher activity compared to SIF-DM and SIF-WM. This difference can be attributed to taro being a prominent source of antioxidant compounds and containing various biologically active phytoconstituents, such as flavonoids, sterols, and glycosides. ^45^

### Rheological analysis

*Pasting curves*

**Figure 2.**
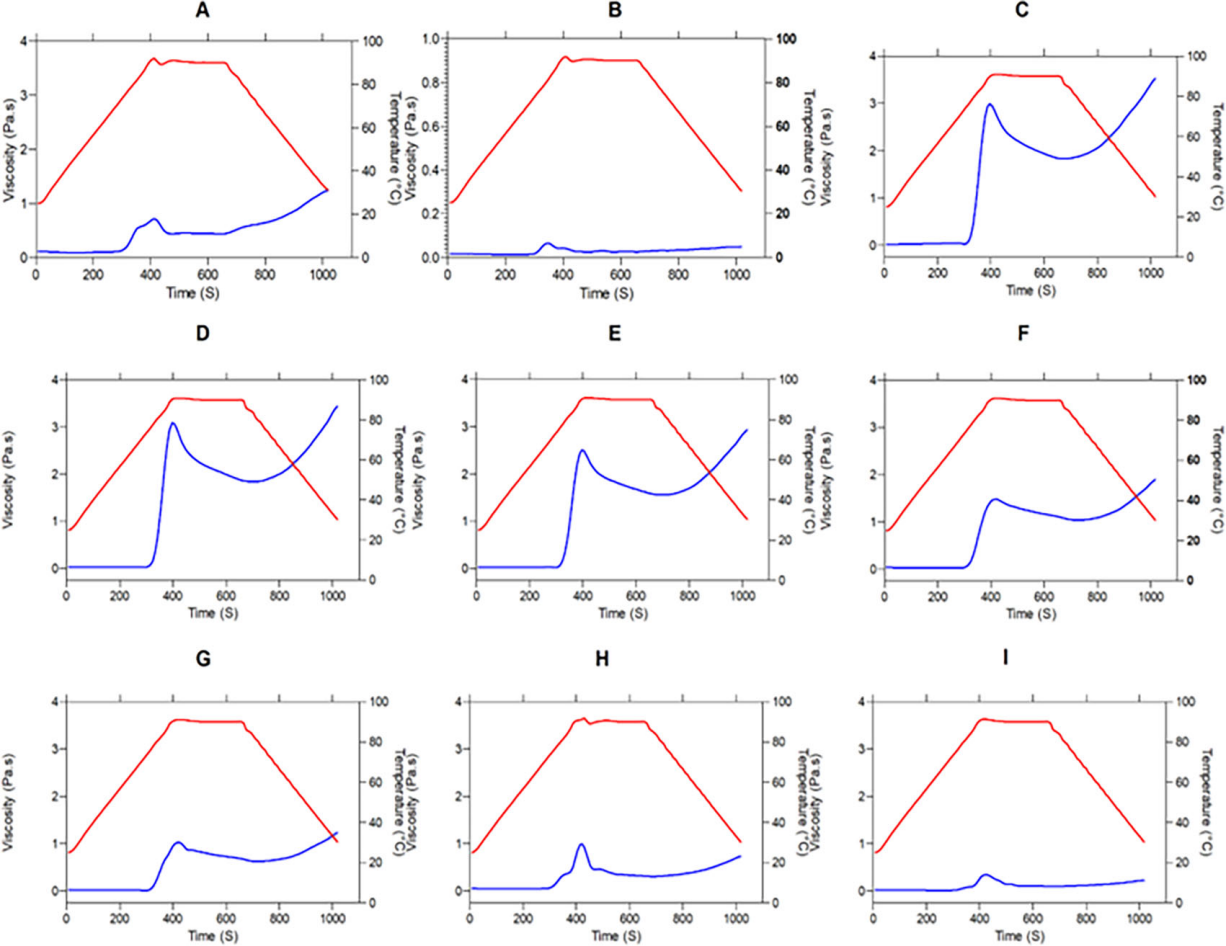
Pasting properties of flour blends. The graphs show the flow profile behavior of: (A) SIF-DM; (B) SIF-WM; (C) TF; (D) 25%SIF-DM:75%TF; (E) 25%SIF-WM:75%TF; (F) 50%SIF-DM:50%TF; (G) 50%SIF-WM:50%TF; (H) 75%SIF-DM:25%TF; (I) 75%SIF-WM:25%TF. The acronym (TF) refers to taro flour; (SIF-WM) sacha inchi flour by wet method and (SIF-DM) sacha inchi flour by defatted method.

Figure 2 shows the viscosity profile of the different flour blends, where the rheological behavior during the heating and cooling phases can be observed. Samples with a higher taro (TF) content exhibited the highest viscosity peaks due to the presence of starch. Taro contains 85.42% carbohydrates, primarily represented by starch. ^48^ Starch granules are not soluble in cold water due to the strong hydrogen bonds that hold the starch chains together. Therefore, when starch is heated in excess water above the gelatinization temperature, it undergoes an order-disorder phase transition known as gelatinization. ^21^ This process is associated with water diffusion into the granule, its absorption by the amorphous region, hydration, and radial swelling of the starch granules, leading to an increase in viscosity. ^13^ As shown in Figure 2 (C, D, E, F), the higher the presence of a starch source, the higher the viscosity peak. This positive correlation between starch content and viscosity peak is consistent with research on various commercial flours from cereals, pseudocereals, roots, and legumes. ^49^

When comparing sacha inchi flours obtained using the dry method (SIF-DM) and the wet method (SIF-WM), it is observed that the flour obtained by the dry method (SIF-DM) exhibits higher viscosity, as seen in Figure 2 (A, B, H, I). This is because, in this process, the defatting of the flour allows for better interaction between water, proteins, and starch ^22^; specifically, SIF-DM flour contains 72.6% protein (see Table 1), which promotes the formation of a more viscous structure by interacting with water.

On the other hand, the particle size of the flour obtained through the wet method (sieved with a standard mesh of 425 to 600 microns) is larger, preventing further size reduction due to the high lipid content. This difference in particle size may also influence the rheological properties of the resulting flour. In this context, the presence of endogenous lipids in taro flour forms a protective layer around carbohydrates and proteins, preventing efficient water absorption and limiting starch swelling during the pasting process. ^50^

**Figure 3.**
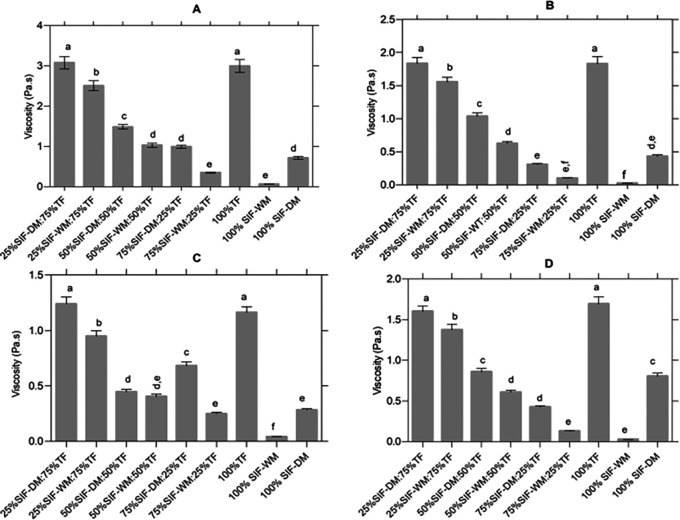
Effect of the different blends on the curves of the pasting curves. The data represent the significant differences in the values of: (A) viscosity peak [Pa.s]; (B) Trough [Pa.s]; (C) Breackdown [°C] and (D) setback [Pa.s]. The acronym (TF) refers to taro flour; (SIF-WM) sacha inchi flour by wet method and (SIF-DM) sacha inchi flour by defatted method.

Next, Figure 3 presents the statistical analysis of the pasting curve properties. In graph 3A, significant differences in viscosity peaks among the different blends are shown. Samples with higher TF content exhibit the highest viscosity peaks due to the presence of starch. This behavior is attributed to the greater amount of water absorbed and the subsequent swelling of starch granules during heating.

Figure 3B shows the behavior of the samples before the onset of retrogradation, that is, the viscosity drop occurring at the end of the constant temperature phase before cooling begins. Samples with higher starch content and lower protein content exhibited a more pronounced viscosity drop. This behavior occurs because once starch reaches its maximum expansion capacity, it releases retained water and solubilizes amylose and amylopectin fractions. Subsequently, during cooling, these macromolecules hydrate individually in excess water and reorganize into a structure similar to the original one. ^51^

Figure 3C presents the significant differences in the breakdown parameter. Breakdown, or stability, is defined as the difference between the peak viscosity and the trough in the constant temperature section, reflecting the disintegration of starch granules at a maintenance temperature of 95 °C under continuous shear. Lower breakdown values indicate greater shear resistance. ^53^ In this study, samples with higher starch content showed lower stability, while those with lower starch content exhibited greater stability. This behavior can be explained by the properties of starch, as during heating, low-molecular-weight amylose separates from the starch granule, causing it to collapse at a constant temperature as the amorphous portion is dispersed, resulting in a decrease in viscosity. ^54^

Finally, Figure 3D presents the analysis of the setback parameter. The setback variable indicates the degree of retrogradation and reorganization of starch molecules during the cooling process, defining the reabsorption of soluble starch polymers and insoluble granular fragments during the cooling phase. ^55^ According to the results, blends with higher TF content exhibited higher setback values, which is due to the higher amylopectin content in taro starch (66% amylopectin versus 34% amylose). ^56, 57^

*Flow profile analysis*

**Figure 4.**
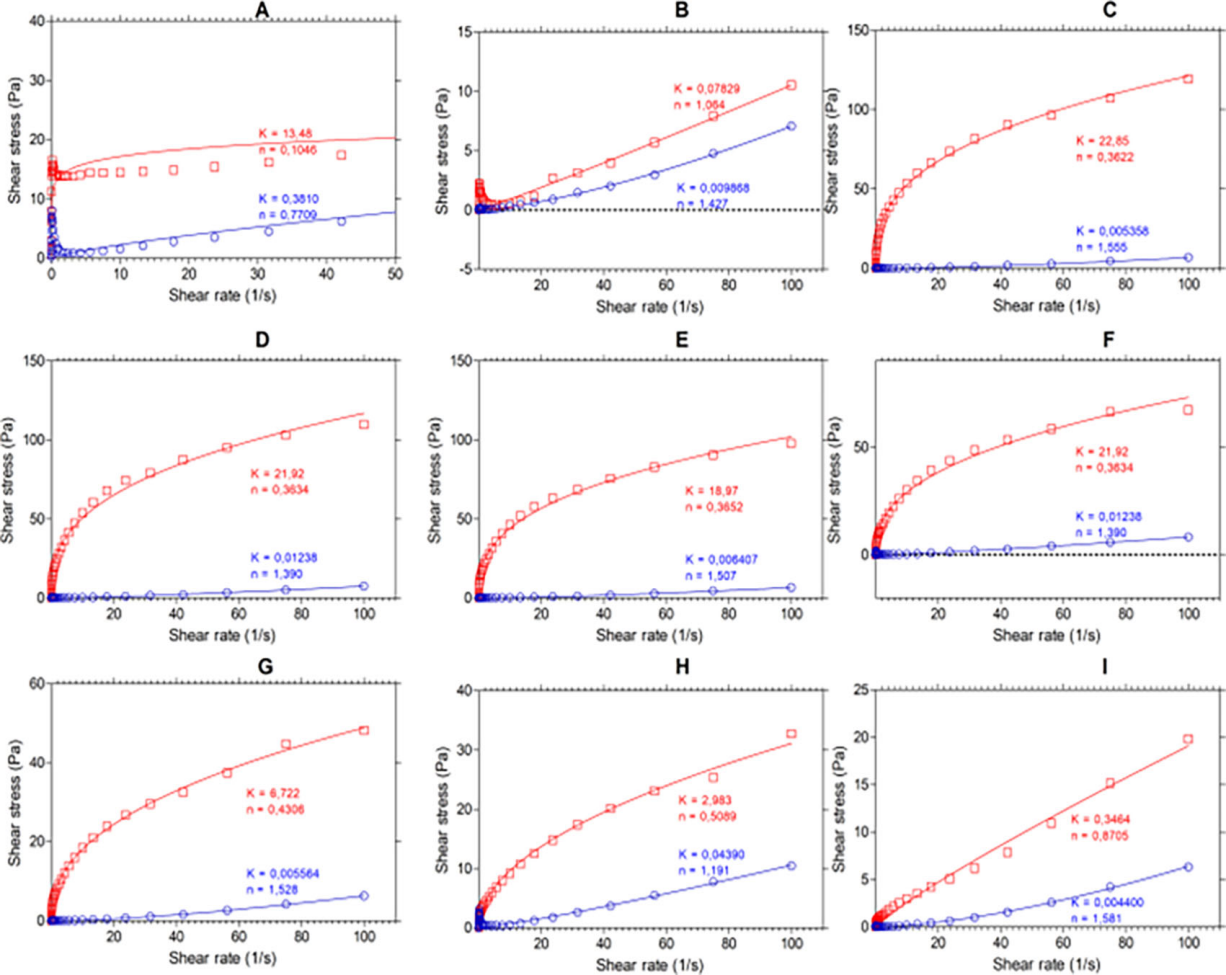
Flow profile of flour blends. The graphs show the flow profile behavior of: (A) SIF-DM; (B) SIF-WM; (C) TF; (D) 25%SIF-DM:75%TF; (E) 25%SIF-WM:75%TF; (F) 50%SIF-DM:50%TF; (G) 50%SIF-WM:50%TF; (H) 75%SIF-DM:25%TF; (I) 75%SIF-WM:25%TF. The acronym (TF) refers to taro flour; (SIF-WM) sacha inchi flour by wet method and (SIF-DM) sacha inchi flour by defatted method.

Figure 4 shows the results of the flow analysis with the viscosity behavior before heating (blue line) and after heating (red line) under stress conditions (shear stress, Pa) and shear rate (s ^-1^). We observed the coefficients obtained by regression of the power model, where “n” is the flow index and “K” is the consistency index. According to the consistency index, we see that “K” increases in all samples except for SIF-WM, because of the viscosity gain, being “n” and “K” inversely proportional parameters.

Regarding the flow index, we observed the behavior of the samples with the highest starch content TF were those that presented the greatest change in “n”, decreasing their flow index represented by the gain in viscosity attributed to the behavior of the starch in taro.

In addition, all the mixtures, except for SIF-DM, showed a value of “n” > 1 before heating, suggesting that they exhibit dilatant fluid behavior. In water and food starch suspensions, dilatant or shear-thickening behavior is related to the initial stiffness of starch granules, which resist shear, and to the high concentration of solids, causing particle swelling. ^58^ SIF-DM, however, had an “n” value of 0.7709, indicating a pseudoplastic fluid behavior.

For the mixture’s behavior after heating, all the blends, except for SIF-WM, presented an n < 1, demonstrating a behavior of a pseudo-plastic fluid, *i.e.,* that changes in temperature affect these mixtures behavior. This behavior occurs because, in the gelatinization process, the starch granules break, releasing amylose to the aqueous medium and, on cooling, these amylose chains align, forming networks that form gels or viscous suspensions. ^21^ The n values for the SIF-WM were reduced to 1.064, exhibiting Newtonian behavior. Newtonian flow indicates that viscosity is independent of shear rate. García-Parra et al. ^58^ suggest that these flours could be suitable for formulating products such as beverages, where improving the nutritional content is required without affecting viscosity during shearing.

*Viscoelastic properties - Temperature sweeping*

**Figure 5.**
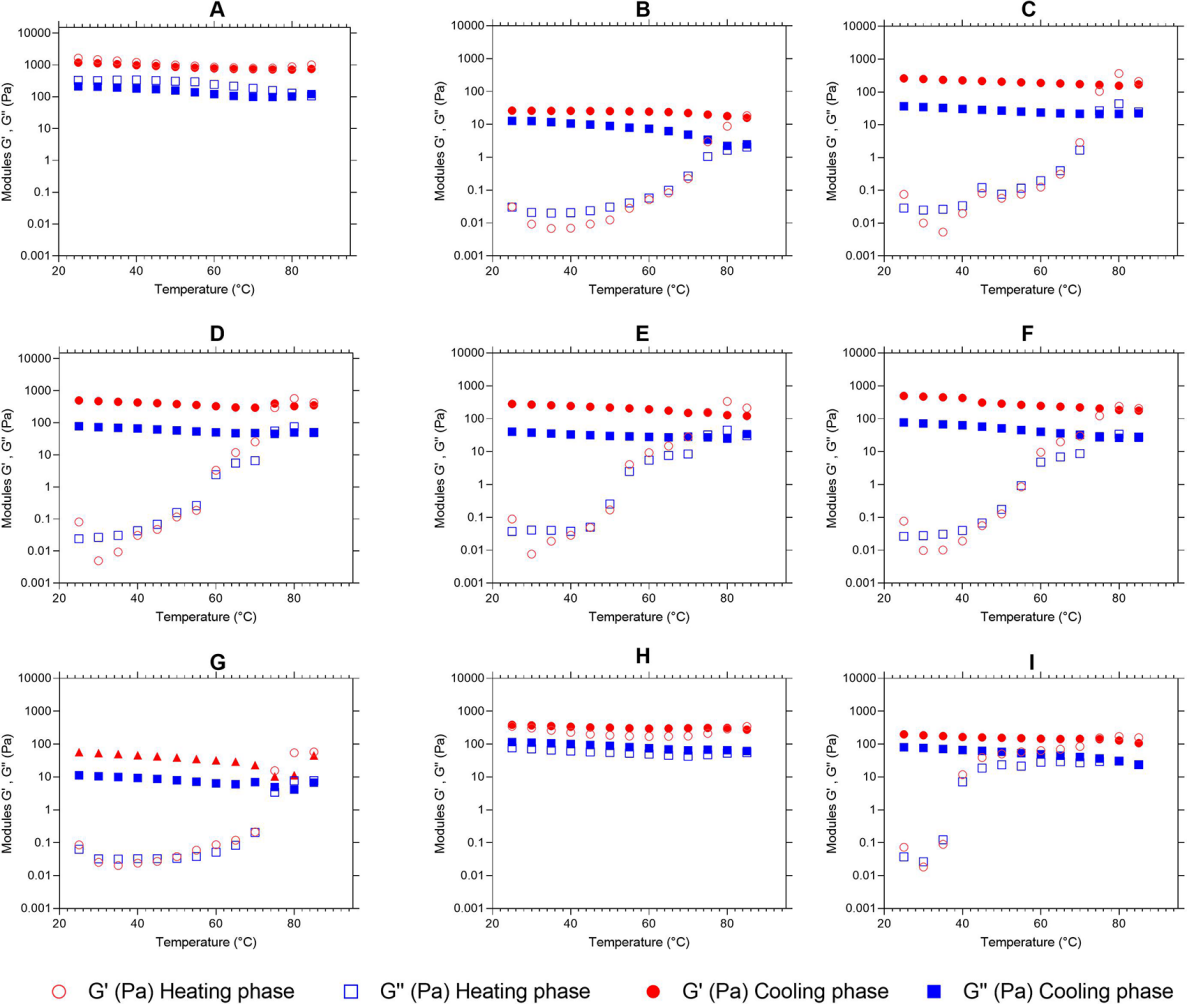
Temperature sweep, G′ and G″ modules of the flour blends. The G′ (red color) and G″ (blue color) modules are presented for: (A) SIF-DM; (B) SIF-WM; (C) TF; (D) 25%SIF-DM:75%TF; (E) 25%SIF-WM:75%TF; (F) 50%SIF-DM:50%TF; (G) 50%SIF-WM:50%TF; (H) 75%SIF-DM:25%TF; (I) 75%SIF-WM:25%TF. The acronym (TF) refers to taro flour; (SIF-WM) sacha inchi flour by the wet method and (SIF-DM) sacha inchi flour by the defatted method.

Figure 5 presents the moduli G′ (red color) and G″ (blue color) as a temperature function (°C). We observe the behavior of the elastic and viscous moduli for each mixture, evidencing an increase in the moduli as the temperature sweeps are performed. Except for graphs “ 5A” and “ 5H”, the mixtures behavior during heating is similar, since the two moduli increase their value, decreasing the difference between them and achieving a crosslinking, where the material ceases to have a viscous character, *i.e.,* it stops being liquid and becomes elastic properties characteristic of a solid. This behavior is characteristic of materials undergoing liquid-solid phase transformations. When the molecules gain weight, in this case, when the starch granules interlock, the loss modulus G″ decreases and the storage modulus G′ increases, reaching a point of equilibrium that is its material change of nature, its solidification.

There is a progressive lixiviation of amylose in the swollen granules, where gel-like linkages are formed. Researchers have reported the effect of amylose to increase the firmness of gels during cooling as one of the initial causes of gel firmness. Thus, during the cooling stage, the moduli stay constant with the predominance of the elastic modulus. ^59^

The behavior of the flour blends is shown in Figure 5A and 5H, which correspond to 100% SIF-DM and 75% SIF-DM: 25% TF, respectively, shows that the G′ and G″ moduli remain constant both during the temperature increase and during subsequent cooling, thus presenting greater stability to temperature variations. This behavior responds to the low starch content of these samples, since starch is key for the viscoelastic behavior of the flours, due to the interaction with water, generating absorption and swelling of the granule and, consequently, greater viscoelasticity. SIF-DM and 75% SIF-DM:25% TF blends did not present a gel point, however, the other blends presented a gel-like behavior, highlighting that the 50% SIF-DM:50% TF and 25% SIF-DM:75% TF blends presented the most elastic gel at a gel temperature of 60°C in both cases, showing similar viscoelastic characteristics.

We suggest an optimum level of protein inclusion represented by sacha inchi between 25 and 50%, where the consistency of the gel increases and mixtures prepared above this range do not present a gel point, while lower values will have a more liquid behavior.

### Microbiological analysis

Table 3 shows the results of the microbiological analysis performed on the three types of flour. For the microbiological analysis, we considered the NTC 6069 ^60^ standard for quinoa flours and NTC 267 ^61^ for wheat flour.

**Table 3.** Microbiological analysis

Microorganism	Specification (log UFC/g)	Counts (log UFC/g)		
		TF	SIF-DM	SIF-WM
Total mesophilic aerobic count	5.47	4.21 ± 0.09	4.32 ± 0.09	3.94 ± 0.05
*Staphylococos* count	<2	2.38 ± 0.09	1.52 ± 0.10	2.44 ± 0.08
Molds and yeasts count	3.7	1.51 ± 0.10	1.60 ± 0.08	2.02 ± 0.06
Total coliform count	<1	Absence	Absence	Absence
*Salmonella* in 25g	Absence	Presence	Absence	Absence

The acronym (TF) refers to taro flour; (SIF-WM) sacha inchi flour by wet method and (SIF-DM) sacha inchi flour by defatted method.

**Figure 6.**
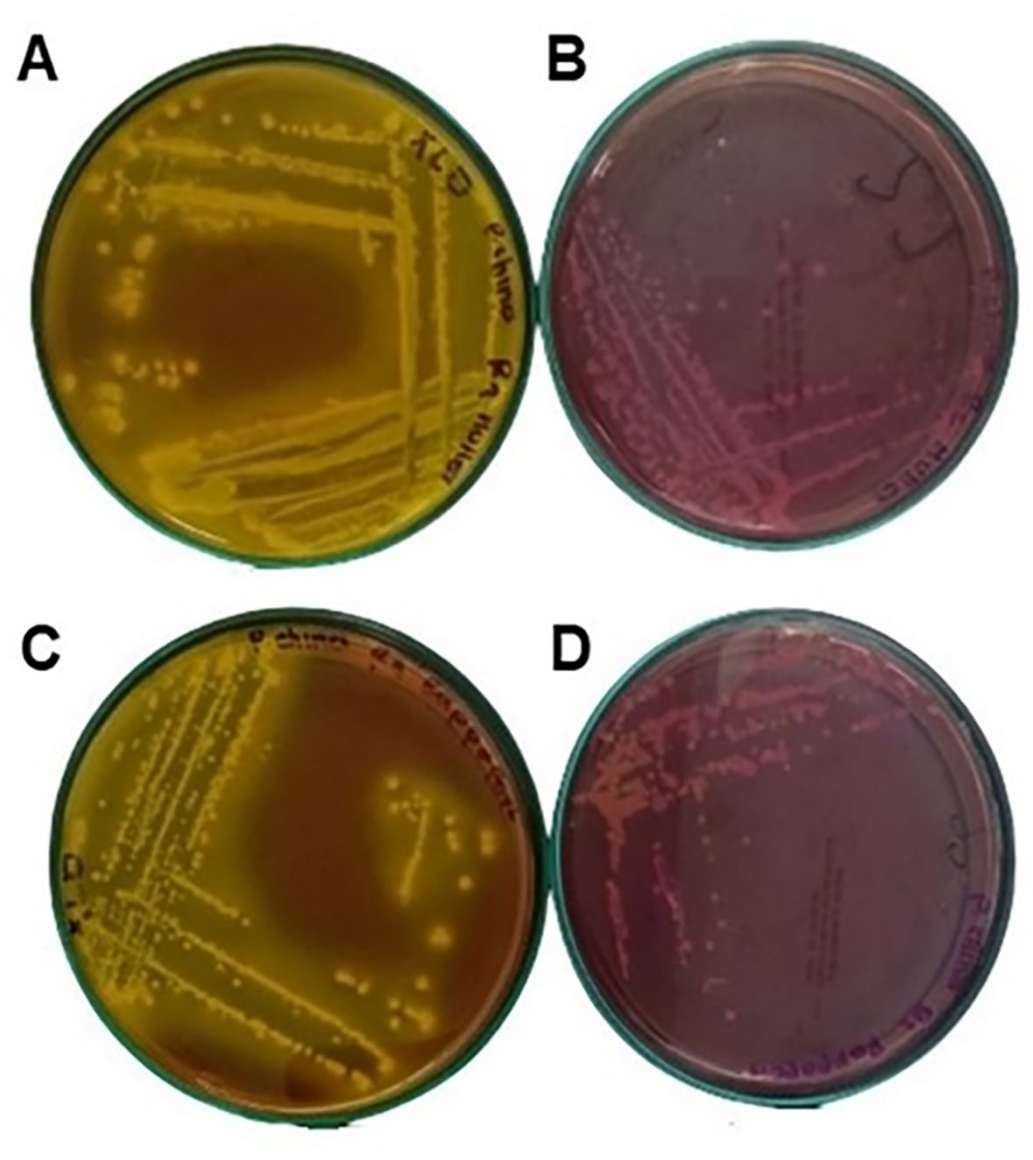
Colonies seeding taro flour sample on XLD agar after 24 h. A: MKTTn broth, C: RVA broth, seeding of taro flour sample on Salmonella-Shigella agar B: MKTTn broth, D: RVA broth.

As can be seen in Table 3, the flours comply with the quality specifications required by Colombian regulations; however, as shown in Figure 6, *Salmonella spp.* is present in the taro flour., possibly due to the persistence of this type of microorganism. Drying this flour at 60 °C preserves certain bioactive compounds but is not sufficient to eliminate it. Larsen et al. ^17^ indicate that these types of pathogens develop resistance to disinfectants and temperature by forming biofilms, which protect them and allow them to persist on equipment, utensils, and surfaces, leading to food contamination. Additionally, flours such as taro may become contaminated during harvesting or transportation.

Table 2 shows that in foods such as sacha inchi flour and taro flour, where water activity is below 0.85, *Salmonella spp.* and other pathogenic bacteria, such as Staphylococcus, can survive in a viable but non-culturable state for extended periods due to their increased resistance to thermal processes. However, although microorganism growth is not sustained at low water activities, foodborne bacteria and fungi can easily contaminate the flour and survive for extended periods with a reduced reproduction rate. ^24^

## Conclusions

Mechanical defatting stood out as the most efficient method for obtaining high-protein flours from sacha inchi. However, this process was observed to reduce the preservation of bioactive compounds, decreasing antioxidant activity and total polyphenol content compared to the wet milling method.

The viscosity profile evaluation determined that the starch present in taro provides good viscosity characteristics, making it an important ingredient for the development of foods such as creams, pastes, sauces, among others. These properties are particularly useful in foods that require a heating process to allow starch gelatinization and, therefore, viscosity development. However, mixtures with an excess of taro have a higher rate of retrogradation, which represents a disadvantage for food stability during storage.

Through the viscoelasticity analysis (temperature sweep), it was determined that an inclusion level of defatted sacha inchi flour between 25% and 50% results in a stable and consistent gel, which could be utilized in various food applications.

The drying temperature of taro at 60 °C preserves polyphenols and antioxidant activity at significant levels; however, it does not allow the inactivation of bacteria such as *Salmonella spp.*, which has shown resistance to thermal treatment in recent years.

The development of foods using gluten-free and underutilized raw materials, such as taro and sacha inchi, which easily adapt to regions facing food security challenges, represents a promising alternative for offering the market highly nutritious and gluten-free products.

Future research can formulate foods from sacha inchi flour, which will allow obtaining high protein foods, responding to the trend of obtaining high protein foods from vegetable sources.

*Salmonella spp.* contamination was present in the taro flour. In response, the facilities and equipment were washed and disinfected with a different product than usual, and again *Salmonella spp.* analyses were performed to guarantee its elimination.

## Data Availability

Zenodo: Article_sacha. https://doi.org/10.5281/zenodo.7582202. ^63^ This project contains the following underlying data:
• Flow profile analysis: folder containing data on the flow analysis of flours in different mixes. • Pasting folder: containing curves and statistical data of pasting properties • Viscoelasticity folder: containing curve data and statistical analysis of analyzed viscoelastic properties. Zenodo: Article_sacha. https://doi.org/10.5281/zenodo.7582202. ^63^ This project contains the following extended data:
• Protocols on proximate and microbiological analysis Data are available under the terms of the Creative Commons Attribution 4.0 International license (CC-BY 4.0).
