# Supplementary material for: Characterization of sacha inchi (Plukenetia volubilis) and taro (Colocasia esculenta) flours with potential application in the preparation of both gluten-free and high protein foods
Source: F1000Res. 2025 Mar 7;12:378. Originally published 2023 Apr 6. [Version 4] doi: 10.12688/f1000research.130394.4 (PMC11809645; doi:10.12688/f1000research.130394.4)
Supplement: Peer review history

# Reviewer report: Reviewer response for version 4

**DOI:** 10.5256/f1000research.177992.r369936

**Published:** 2025-04-02

**Authors:** Lorena Sciarini

**Affiliations:** 1 Universidad Nacional de Cordoba, Córdoba, Cordoba, Argentina

The authors have made most of the requested revisions. However, some issues remain unresolved, and new ones have arisen, especially regarding SIF composition and rheological properties.

**Pp 2. Abstract.** 7.47 mg GAE/g corresponds to FPP content, not antioxidant activity.

**Pp 5.** Please, do not start sentences with a number. Check throughout the manuscript.

**Pp 5. Protein determination.** Which factor was used for protein calculation?

**Pp 6.** Please change “Fiber” to “Crude fiber.”

**Table 1.** “The lipids results were close, 9.84% for the SIF-DM and 8.87% for SIF-WM.” This result is not intuitive. While DM mainly extracts lipids, WM extracts hydrophilic compounds. Why do they have the same lipid content?

 In general, the composition of SIF is striking, especially in terms of protein and lipid content (since carbohydrates are calculated by difference, their values are relative). The difference in protein concentration may be due to partial solubilization during the WM process. The difference from the almond is also noteworthy. Another remarkable aspect is the (small) difference in lipid content. I suggest the authors to review these values.

**Pp 9.** Please unify terminology throughout the text: dry method, defatted method, mechanical pressing.

**Pp 9.** “On the other hand, it is likely that carbohydrates are solubilized in the water used in the wet method, contributing to their higher content in SIF-WM.” This sentence is contradictory. Please revise.

**Pp 10.** Change “by 43” to “by Sánchez-Rivera 43.”

**Figure 3B** seems to present Trough viscosity values. Please check.

**Pp 11.** Change “and solubilizes amylose and amylopectin fractions” to “and amylose partially leaches out from granules” (the term “solubilizes” is not correct in this context).

**Pp 11.** “Reorganize into a structure similar to the original one” is not accurate. Starch gelatinization is an irreversible phenomenon. During cooling and storage, amylose and amylopectin do reorganize into partially crystalline structures, but these are completely different from the original starch granules.

**Pp 11.** “This behavior can be explained by the properties of starch, as during heating, low-molecular-weight amylose separates from the starch granule, causing it to collapse at a constant temperature as the amorphous portion is dispersed, resulting in a decrease in viscosity.” In general, breakdown positively correlates with peak viscosity: the more water the starch granule absorbs, the more fragile it becomes.

**Pp 11.** “According to the results, blends with higher TF content exhibited higher setback values, which is due to the higher amylopectin content in taro starch (66% amylopectin versus 34% amylose).” It is unclear what is being compared to what. The starch source is TF, which always has the same AM-AP ratio. Additionally, setback is related to amylose retrogradation, not amylopectin. Finally, in this study, the higher setback is likely related to the higher starch content.

**Figure 4.** Please clarify the image references (symbols) in the figure caption.

**Figures 5A and 5H.** The relatively high modulus values from the beginning of the test at room temperature are remarkable. Please comment.

**Conclusion.** “Mechanical defatting stood out as the most efficient method for obtaining high-protein flours from sacha inchi. However, this process was observed to reduce the preservation of bioactive compounds, decreasing antioxidant activity and total polyphenol content compared to the wet milling method.” This seems to be the opposite, as the FPP and ABTS values are higher in SIF-DM than in SIF-WM.

Is the work clearly and accurately presented and does it cite the current literature?

Yes

If applicable, is the statistical analysis and its interpretation appropriate?

Yes

Are all the source data underlying the results available to ensure full reproducibility?

Yes

Is the study design appropriate and is the work technically sound?

Yes

Are the conclusions drawn adequately supported by the results?

Partly

Are sufficient details of methods and analysis provided to allow replication by others?

Yes

Reviewer Expertise:

Gluten free bakery, starch properties, cereal properties

I confirm that I have read this submission and believe that I have an appropriate level of expertise to confirm that it is of an acceptable scientific standard, however I have significant reservations, as outlined above.

# Reviewer report: Reviewer response for version 3

**DOI:** 10.5256/f1000research.174336.r349309

**Published:** 2024-12-27

**Authors:** Lorena Sciarini

**Affiliations:** 1 Universidad Nacional de Cordoba, Córdoba, Cordoba, Argentina

Pp1. Abstract. “The ABTS radical method, which reacts with the phenolic compounds of the food matrix, using Trolox as a standard.” Please review the wording (the sentence is incomplete).

 Pp 2. Does "Salmonella" correspond to a specific species? Please revise (it could also be Salmonella sp. or Salmonella spp.).

 Pp2. It is suggested to change “According to Ref. 1” to “According to Wang et al.¹.” Consider applying this modification throughout the text.

 Pp 2. “According to Ref. 1, by 2050, if governance allows for debate and equity in global food and if food demand is very high, driven by rapid population growth, a high intensity of land use will be required to meet the demand for food”. The wording is unclear. Please revise.

 Pp 4. Please, replace “mNicroorganisms” with “microorganisms”

 Pp4. “this, in order to grind the grain. Obtaining a milky and homogeneous” Please revise the punctuation (I understand the period is not appropriate).

 Pp4. Deffating method. Was the extraction cake dried? How was it milled afterward? Was it sieved? Please complete the methodology.

 Pp5. For the proximate composition, please specify which AOAC method was used in each case.

 Pp6. Ref. 20 is incomplete in the References section. Please review all citations.

 Pp 6. Polyphenols. “Finally, the supernatant mixture was made up to 20 mL using distilled water and the absorbance was measured in the spectrophotometer”. Which colorimetric technique was used? Folin-Ciocalteu? Please clarify.

 Pp6. ABTS. “In a test tube 4mL of ABTS.* (2,20-Azino-bis(3-ethylbenzthiazoline-6-sulfonic acid), solution was added and to start the reaction 135 μL standard solution and vortexed for 5 seconds.” Please revise the wording.

 Pp6. Flow profile. Which geometry was used?

 Pp7. Fungi and yeast. “30°” with “30 °C”.

 Pp8. Results and discussion. Table 1. The wet method of obtaining Sacha inchi flours results in the solubilization of hydrophilic compounds, such as proteins. This causes the concentration of other components to increase (particularly carbohydrates, according to the authors' findings). However, it is striking that the lipid concentration is so similar, given that the mechanical method specifically extracts the oil. What is the reason for this similarity?

 Pp8. “As for carbohydrate content, SIF-DM showed a lower percentage of carbohydrates compared to other extraction methods, due to the fact that in the oil extraction process, not only fat is removed but also some solids such as proteins, minerals, and certain carbohydrates (see Figure 1B). These components may become trapped in the oil or be eliminated with the byproduct generated during extraction. Additionally, it is likely that some soluble carbohydrates dissolve or are removed along with the oil during mechanical extraction”. It is more likely that carbohydrates are solubilized in the water used in the wet method rather than in the oil, although some carryover may occur. It is worth exploring the fact that the wet method actually has a higher carbohydrate content because, proportionally, it has fewer proteins, leading to a concentration of carbohydrates (which are calculated by difference).

 Pp9. Was the proximate composition of the flour blends measured or calculated?

 Pp9. Effect of processing on FPP and the antioxidant activities. Please do not repeat in the text the values already presented in Tables/Figures. Verify this throughout the manuscript. Also, check, as "taro flour presents the highest polyphenol content of 7.50+/-1.73" does not match the value presented in Table 2.

 Pp 10. “This difference can be explained because taro is a prime source of antioxidant” What is meant by "prime source"?

 Pp10. “… bioactive compounds, which in sacha inchi are present particularly in polyunsaturated essential fatty acids…” ABTS is used to evaluate the radical scavenging capacity of hydrophilic compounds. In fact, the extraction was performed with polar solvents. I find the explanation contradictory.

 Pp11. “to the post hoc Tukey analysis five different groups identified with letters from “a” to “e” are classified. In group “a”, the samples with the highest viscosity (25% SIF-DM:75% TF and 100% TF) with values of 3.08 Pa.s and 2.99 Pa.s respectively, and in group “e”, the samples with the lowest viscosity value (75% SIF-WM:25% TF and 100% SIF-WM) with values of 0.35 Pa.s and 0.64 Pa.s respectively.” This type of expression used throughout the manuscript makes the reading tedious and is repeated with what is presented in graphs/tables. Please simplify by focusing on the statistical differences presented between samples.

 Pp12. In this page, the results are presented in a disorganized manner. It is suggested to first address Figure 2, and then Figure 3.

 Pp12. “According to the above, as shown in Figure 3, the samples that presented a higher viscosity drop are the samples with higher TF content, this is because the crystalline structure (amylopectin) that makes up the granules is altered, when amylose and amylopectin are hydrated individually in excess water, both macromolecules tend to reorganize in a similar structure to the one they had initially.” The authors discuss the minimum viscosity (or trough), but I believe there is some confusion here, as this parameter is not associated with retrogradation but with gelatinization. The granules absorb water up to a maximum, and with shear at high temperature, they break, decreasing viscosity. Retrogradation occurs when the temperature decreases.

 Pp12. “As shown in Figure 3, mixtures with higher inclusion of starch present in TF and less protein contributed by sacha inchi result in more free water for starch molecules to swell and solubilize presenting higher viscosity; on the contrary, at high proportions of protein its interaction with starch granules could stabilize the amylose leaching channel of starch granules, which resulted in lower swelling power and solubility.” It would be useful to consider, in the mixtures, the effect of dilution of the starch present in TF. On the other hand, proteins also develop viscosity, which could explain why TF+SIF-DM has higher viscosity than TF+SIF-WM.

 Pp12. “until the amorphous part is solubilized, decreasing viscosity”. In general, when referring to starch in water, the term "dispersion" is used, not "solubilization."

 Pp12. Flow profile analysis. Please, unify: “flowability index” or “flow index”.

 Pp12. Two consecutive paragraphs begin with "Regarding the Flow index." Please review the wording.

 Pp12. “dilettantish fluid” or “dilatant fluid”?

 Pp13. Viscoelastic properties - Temperature sweep. Figure 5 needs more details. First, the color assigned to each module should be clarified in the figure caption. Second, it should be specified which curve corresponds to the heating phase and which to the cooling phase. Without this information, it is not possible to follow the discussion.

 Pp13. “progressive exudation” by “progressive lixiviation”.

 Pp 14. “where aw is less than 0.85”. Lower?

 Pp 15. Conclusions. “The method that allows for the highest concentration of protein in sacha inchi is mechanical defatting with a protein result of 73%, compared to that obtained by the wet method, which resulted in 31%.” This does not belong in the Conclusion section, but rather in the Results and Discussion section.

 Pp 15. “However, during the production of sacha flour with the mechanical method, bioactive compounds are lost, which is why lower values of total polyphenols and antioxidant activity are obtained, compared to taro flour.” In this sentence, methods for obtaining Sacha Inchi flour are compared, but it is compared with taro flour. Please review the wording.

 Pp 15. “The viscosity profile evaluation determined that the starch contained in taro provides good viscosity characteristics, being this an important ingredient for the development of foods such as creams, pastes, sauces, among others.” This is useful for foods that require a heating process to allow starch gelatinization and hence viscosity development.

 Pp15. “the optimum level of inclusion”. In fact, the optimal level will depend on the food that is being formulated.

 Pp16. “Although taro’s drying temperature of 60 °C maintains polyphenols and antioxidant activity”. We do not know if they "maintain," as it was not compared against other drying temperatures.

 Pp16. “using gluten-free orphan raw materials”. What do authors mean by "orphan"?

Is the work clearly and accurately presented and does it cite the current literature?

Yes

If applicable, is the statistical analysis and its interpretation appropriate?

Yes

Are all the source data underlying the results available to ensure full reproducibility?

Yes

Is the study design appropriate and is the work technically sound?

Yes

Are the conclusions drawn adequately supported by the results?

Partly

Are sufficient details of methods and analysis provided to allow replication by others?

Yes

Reviewer Expertise:

Gluten free bakery, starch properties

I confirm that I have read this submission and believe that I have an appropriate level of expertise to confirm that it is of an acceptable scientific standard, however I have significant reservations, as outlined above.

# Response

**Published:** 2025-02-05

**Authors:** Karen Muñoz

**Affiliations:** University of Cauca, Colombia

Dear Dr. Lorena Sciarini,

 We would like to express our sincere gratitude for the valuable contributions you made during the evaluation process of our article. Your suggestions have been highly enriching, and we have taken them into account in order to enhance the quality and clarity of our work. Below we

 respond to each of the suggestions made.

**Pp1.** **Abstract. “The ABTS radical method, which reacts with the phenolic compounds of the food matrix, using Trolox as a standard.” Please review the wording (the sentence is incomplete).**

**Response: **Thank you for your observation. We have reviewed and corrected the wording of the sentence to make it clearer and more complete, while also complementing the main idea. The revised version is as follows: “ **Methods**: Proximal tests were conducted to determine basic chemical composition, quantification of free polyphenols, antioxidant activity using the

 ABTS* radical method (2,2′-azino-bis (3-ethylbenzothiazoline-6-sulfonic acid)) with Trolox as a standard, and rheological analyses, including pasting curves, flow profiles, and viscoelastic properties. Microbiological characterization of the flours was also performed.”

**Pp 2. Does "Salmonella" correspond to a specific species? Please revise (it could also be Salmonella sp. or Salmonella spp.).**

**Response: **We have reviewed the entire document and replaced ' *Salmonella*' with the correct designation *'Salmonella spp.*' to refer to the corresponding species.

**Pp2. It is suggested to change “According to Ref. 1” to “According to Wang et al.¹.” Consider applying this modification throughout the text.**

**Response: **Thank you for your observation. We have taken your suggestion into account and have adjusted the referencing format throughout the text accordingly

**Pp 4. Please, replace “mNicroorganisms” with “microorganisms”**

**Response: **Thank you very much for the observation. It has been replaced with the appropriate term “microorganisms”.

**Pp4. “this, in order to grind the grain. Obtaining a milky and homogeneous” Please revise the punctuation (I understand the period is not appropriate).**

**Response: **The suggestion was taken into account, and the wording was improved to enhance the clarity of the text. The revised version is as follows: “The sacha inchi flour

 obtained by the wet method (SIF-WM) was prepared as follows: initially, wet grinding was carried out using a blender (Oster, BLST 4655, Colombia) with a capacity of 1.25 L. For this process, sacha inchi seeds were mixed with water in a 1:3 ratio to ensure effective grinding. This resulted in a milky and homogeneous suspension. Subsequently, a cloth filter was used to separate the insoluble extract (cake) from the water-soluble extract (milk). The cake was

 then dried in an oven at 65 °C for 3 hours. Finally, once dehydrated, it was immediately packaged in a polyethylene bag with a hermetic seal for preservation [18].”

**Pp4. Deffating method. Was the extraction cake dried? How was it milled afterward? Was it sieved? Please complete the methodology.**

**Response: **La requested information was completed and the text's wording was improved. The modified version is as follows: “The defatted sacha inchi flour (SIF-DM) was obtained as follows: the sacha inchi kernels were placed in the hopper of an automatic touchscreen oil extraction press (CGLDENWALL, model K28, Shanghai, China), as shown in Figure 1. The process was carried out at a temperature of 124 °C. During the extraction, a screw conveyor pushed the material into the main pressing cylinder. As it moved forward, the design of the screw, with decreasing pitch and spiral depth, reduced the available volume in the chamber,

 subjecting the material to high pressure and friction against the closed bottom wall of the cylinder. This facilitated efficient oil extraction, with the oil flowing through the cylinder's orifices, while the residual material, known as cake, was expelled through the discharge nozzle. Subsequently, the cake was ground using an electric mill (Quaker City Mill, model 4-E, Philadelphia) and sieved through a system with a No. 30 mesh to achieve uniform particle size. Finally, the resulting flour was packaged in polyethylene bags with airtight seals for preservation.”

**Pp5. For the proximate composition, please specify which AOAC method was used in each case.**

**Response: **The provided suggestion was taken into account, and in response, the AOAC method used for each compositional analysis was included in each case.

*Protein: “*For the determination of protein content, the protocol established by AOAC 968.06 was followed.”

*Fiber: *“The fiber content was analyzed according to the AOAC 962.09 protocol.”

***Ash:*** **“ **The ash content was determined following the protocol established by AOAC 942.05.”

**Pp6. Ref. 20 is incomplete in the References section. Please review all citations.**

**Response: **All references have been reviewed and completed where necessary.

**Pp 6. Polyphenols. “Finally, the supernatant mixture was made up to 20 mL using distilled water and the absorbance was measured in the spectrophotometer”. Which colorimetric technique was used? Folin-Ciocalteu? Please clarify.**

**Response: **The raised concern was taken into account, and the wording was complemented and improved to ensure greater clarity, resulting in the following enhanced versión: “For the second extraction, 8 mL of 70% acetone with 1% formic acid (70 mL acetone + 30 mL water + 1 mL formic acid) was added to the pellet from the first extraction. The pellet was shaken in the solution for 25 minutes in the shaker at 200 RPM at room temperature. Subsequently, the

 samples were centrifuged at 3500 RPM for 5 minutes at room temperature, and the second supernatant was combined with the first supernatant and shaken. The resulting mixture was made up to 20 mL using distilled water. Next, the measurement was carried out using the Folin-Ciocalteu method. 20 µL of the extract was taken and combined with 900 µL of Folin reagent, vortexed, and then 600 µL of NaHCO3 was added, shaking again to ensure proper dissolution. The reaction was allowed to rest, and finally, the absorbance was measured at 765 nm in the spectrophotometer (Thermo Scientific, Genesys 10S UV VIS).”

**Pp6. Ref. 20 is incomplete in the References section. Please review all citations.**

**Response: **All references have been reviewed and completed where necessary.

**Pp 6. Polyphenols. “Finally, the supernatant mixture was made up to 20 mL using distilled water and the absorbance was measured in the spectrophotometer”. Which colorimetric technique was used? Folin-Ciocalteu? Please clarify.**

**Response: **The raised concern was taken into account, and the wording was complemented and improved to ensure greater clarity, resulting in the following enhanced versión: “For the second extraction, 8 mL of 70% acetone with 1% formic acid (70 mL acetone + 30 mL water + 1 mL formic acid) was added to the pellet from the first extraction. The pellet was shaken in the solution for 25 minutes in the shaker at 200 RPM at room temperature. Subsequently, the

 samples were centrifuged at 3500 RPM for 5 minutes at room temperature, and the second supernatant was combined with the first supernatant and shaken. The resulting mixture was made up to 20 mL using distilled water. Next, the measurement was carried out using the Folin-Ciocalteu method. 20 µL of the extract was taken and combined with 900 µL of Folin reagent, vortexed, and then 600 µL of NaHCO3 was added, shaking again to ensure proper dissolution. The reaction was allowed to rest, and finally, the absorbance was measured at 765 nm in the spectrophotometer (Thermo Scientific, Genesys 10S UV VIS).”

Pp6. ABTS. “In a test tube 4mL of ABTS.* (2,20-Azino-bis(3-ethylbenzthiazoline-6-sulfonic acid), solution was added and to start the reaction 135 μL standard solution and vortexed for 5 seconds.” Please revise the wording.

**Response: **The suggestion made was taken into account, and the wording was improved, resulting in the following: “In a test tube, 4 mL of ABTS.* solution (2,2′-azino-bis(3-ethylbenzothiazoline-6-sulfonic acid)) was added, followed by 135 μL of the standard solution, and it was vortexed for 5 seconds. For the reagent blank, a mixture of 4 mL of 4.5 acetate buffer and 135 μL of ethanol was used. The zero point was prepared with 4 mL of ABTS solution and 135 μL of ethanol. The tube was then capped and allowed to rest for exactly 30 minutes before measuring the absorbance in the spectrophotometer (Thermo Scientific, Genesys 10S UV VIS) at a wavelength of 729 nm.”

**Pp6. Flow profile. Which geometry was used?**

**Response: **The question raised was answered, and the wording was improved. The modified version is as follows: “The flow properties were determined following the methodology outlined by Polo and Roa [21], with some modifications. An AR1500 rheometer (TA Instruments, New Castle, USA) was used for this study, and measurements were taken using cylindrical geometry. The average viscosity was determined at 25 °C for 12 minutes, with the shear rate

 increasing in 4 steps as follows:…)

**Pp7. Fungi and yeast. “30°” with “30 °C”.**

**Response: **Thank you for the observation, the corresponding correction has already been made.

**Pp8. Results and discussion. Table 1. The wet method of obtaining Sacha inchi flours results in the solubilization of hydrophilic compounds, such as proteins. This causes the concentration of other components to increase (particularly carbohydrates, according to the authors' findings). However, it is striking that the lipid concentration is so similar, given that the mechanical method specifically extracts the oil. What is the reason for this similarity?**

**Response: **The similarity in lipid concentration between the flours obtained through the wet and mechanical methods can be explained by the specific characteristics of each process. In the wet method, sacha inchi seeds are mixed with water, and while there is no initial loss of solids, some hydrophilic compounds, such as proteins and carbohydrates, migrate into the

 solvent during the soaking and homogenization stages. During filtration, the cake retains the lipids, while soluble compounds are removed with the liquid byproduct. Additionally, the cake obtained through the wet method does not undergo the compression applied by the mechanical press, meaning the necessary pressure to fully extract the oil is not achieved. As a result, a greater amount of lipids is retained in the final cake from the wet method, which explains the similarity in lipid concentration values between the two methods.

**Pp8. “As for carbohydrate content, SIF-DM showed a lower percentage of carbohydrates compared to other extraction methods, due to the fact that in the oil extraction process, not only fat is removed but also some solids such as proteins, minerals, and certain carbohydrates (see Figure 1B). These components may become trapped in the oil or be eliminated with the byproduct generated during extraction. Additionally, it is likely that some soluble carbohydrates dissolve or are removed along with the oil during mechanical extraction”. It is more likely that carbohydrates are solubilized in the water used in the wet method rather than in the oil, although some carryover may occur. It is worth exploring the fact that the wet method actually has a higher carbohydrate content because, proportionally, it has fewer proteins, leading to**

**a concentration of carbohydrates (which are calculated by difference).**

**Response: **Thank you very much for the observations. We took the suggestions into account, resulting in the following version: ” Regarding carbohydrate content, SIF-DM showed a lower percentage compared to other extraction methods. This can be explained by the fact that during the mechanical oil extraction process, not only fat is removed but also some solids, such as proteins, minerals, and certain carbohydrates, which could become trapped in the oil or be eliminated along with the byproducts generated during extraction (see Figure 1B). On the other hand, it is likely that carbohydrates are solubilized in the water used in the wet method, contributing to their higher content in SIF-WM. Additionally, the higher carbohydrate content observed in SIF-WM could be due to a proportional effect: the reduction in proteins during the wet process increases the relative proportion of carbohydrates, which are calculated by difference. Moreover, the wet method may promote the retention of insoluble

 carbohydrates within the solid matrix, minimizing the loss of hydrophilic compounds and explaining the higher carbohydrate content in SIF-WM.”

**Pp9. Was the proximate composition of the flour blends measured or calculated?**

**Response: **The wording was adjusted based on the observation made. Below, the revised and corrected version is presented: “The study design was based on the type of flour and its inclusion levels in the mixture, considering protein content and carbohydrate percentage as the most relevant factors in each treatment. The proximal composition of the blends was calculated using the individual results obtained from each flour. The analyses showed that the 25% SIF-DM:75% TF blend contained 22.69% protein and 64.83% carbohydrates; the 50% SIF-DM:50% TF blend showed 39.34% protein and 44.23% carbohydrates; and the 75% SIF-DM:25% TF blend reached 55.98% protein and 23.64% carbohydrates. On the other hand, the blends with SIF-WM presented the following values: 25% SIF-WM:75% TF with 12.42%

 protein and 73.47% carbohydrates; 50% SIF-WM:50% TF with 18.80% protein and 61.52% carbohydrates; and finally, the 75% SIF-WM:25% TF blend with 25.17% protein and 49.56% carbohydrates.”

**Pp9. Effect of processing on FPP and the antioxidant activities. Please do not repeat in the text the values already presented in Tables/Figures. Verify this throughout the manuscript. Also, check, as "taro flour presents the highest polyphenol content of 7.50+/-1.73" does not match the value presented in Table 2.**

**Response: **We have taken the suggestions into account and corrected the corresponding values. The modified version is as follows: “Table 2 highlights statistically significant differences in the total polyphenol content among the flours analyzed. Taro flour exhibited the highest polyphenol content, aligning with findings reported by Eleazu et al. [42] and surpassing other food sources such as plantain flour, as noted by [43]. Meanwhile, defatted sacha inchi flour demonstrated a higher polyphenol content compared to the flour obtained through the wet method. This result is likely due to the mechanical extraction process, which involves pressure and temperature, facilitating the release of phenolic compounds, as previously observed [44].”

**Pp 10. “This difference can be explained because taro is a prime source of antioxidant” What is meant by "prime source"?**

**Response: **We aimed to replace the term 'primary source' with a more context-appropriate expression, resulting in the following revision: “Regarding the antioxidant activity measured using the ABTS method, taro flour demonstrated significantly higher activity compared to SIF-DM and SIF-WM. This difference can be attributed to taro being a prominent source of antioxidant compounds and containing various biologically active phytoconstituents, such as flavonoids, sterols, and glycosides [45].”

**Pp10. “… bioactive compounds, which in sacha inchi are present particularly in polyunsaturated essential fatty acids…” ABTS is used to evaluate the radical scavenging capacity of hydrophilic compounds. In fact, the extraction was performed with polar solvents. I find the explanation contradictory.**

**Response: **You are absolutely right; the explanation was contradictory. Therefore, we have

 revised the analysis and discussion of results, resulting in the following versión. “In this study, a drying temperature of 60 °C was used for the taro flour (TF), which allows for achieving the maximum antioxidant activity in taro flours [46]. The SIF-DM showed the lowest antioxidant activity, which can be attributed to the loss of polar hydrophilic compounds, such as polyphenols, during the mechanical extraction. These compounds are the main contributors to

 the radical scavenging capacity measured using the ABTS

**Pp11. “to the post hoc Tukey analysis five different groups identified with letters from “a” to “e” are classified. In group “a”, the samples with the highest viscosity (25% SIF-DM:75% TF and 100% TF) with values of 3.08 Pa.s and 2.99 Pa.s respectively, and in group “e”, the samples with the lowest viscosity value (75% SIF-WM:25% TF and 100% SIF-WM) with values of 0.35 Pa.s and 0.64 Pa.s respectively.” This type of expression**

**used throughout the manuscript makes the reading tedious and is repeated with what**

**is presented in graphs/tables. Please simplify by focusing on the statistical differences presented between samples.**

**Response: **We took the suggestion into account throughout the manuscript and simplified some expressions to make the reading more fluid and improve its quality

**Pp12. In this page, the results are presented in a disorganized manner. It is suggested to first address Figure 2, and then Figure 3.**

**Response: **Thank you for your suggestion. We have organized the results as you recommended and rewritten them to improve clarity.

**Pp12. “According to the above, as shown in Figure 3, the samples that presented a higher viscosity drop are the samples with higher TF content, this is because the crystalline structure (amylopectin) that makes up the granules is altered, when amylose and amylopectin are hydrated individually in excess water, both macromolecules tend to reorganize in a similar structure to the one they had initially.” The authors discuss the minimum viscosity (or trough), but I believe there is some confusion here, as this**

**parameter is not associated with retrogradation but with gelatinization. The granules absorb water up to a maximum, and with shear at high temperature, they break, decreasing viscosity. Retrogradation occurs when the temperature**

**decreases.**

**Response: **The suggestion was taken into account, the information was organized, and the wording was improved, resulting in the following structured idea: “In graph 3.A, significant differences in viscosity peaks among the different blends are shown. Samples with higher TF content exhibit the highest viscosity peaks due to the presence of starch. This behavior is attributed to the greater amount of water absorbed and the subsequent swelling of starch granules during heating.

 Figure 3.B shows the behavior of the samples before the onset of retrogradation, that is, the viscosity drop occurring at the end of the constant temperature phase before cooling begins. Samples with higher starch content and lower protein content exhibited a more pronounced viscosity drop. This behavior occurs because once starch reaches its maximum expansion capacity, it releases retained water and solubilizes amylose and amylopectin fractions. Subsequently, during cooling, these macromolecules hydrate individually in excess water and reorganize into a structure similar to the original one [51].”

**Pp12. “As shown in Figure 3, mixtures with higher inclusion of starch present in TF and less protein contributed by sacha inchi result in more free water for starch molecules to swell and solubilize presenting higher viscosity; on the contrary, at high proportions of protein its interaction with starch granules could stabilize the amylose leaching**

**channel of starch granules, which resulted in lower swelling power and solubility.” It would be useful to consider, in the mixtures, the effect of dilution of the starch present in TF. On the other hand, proteins also develop viscosity, which could explain why TF+SIF-DM has higher viscosity than TF+SIF-WM.**

**Response:** The observation was taken into account, and the text was adjusted as follows: “…Conversely, in blends with a higher protein content, proteins interact with starch granules, stabilizing the amylose leaching channel and reducing both swelling and solubility of starch. This behavior is influenced by the dilution of starch in taro flour (TF), as a lower starch concentration in the blend limits its ability to absorb water and form viscous structures [52]. Additionally, proteins play a crucial role in viscosity development, which explains why TF+SIF-DM blends exhibit higher viscosity compared to TF+SIF-WM blends. In TF+SIF-DM blends, the preservation of functional proteins promotes more effective interaction with water and starch molecules, resulting in higher viscosity. In contrast, in TF+SIF-WM blends, the loss of proteins during the wet extraction process reduces their contribution to viscosity, thereby lowering the rheological performance of the blend.”

**Pp12. “until the amorphous part is solubilized, decreasing viscosity”. In general, when referring to starch in water, the term "dispersion" is used, not "solubilization."**

**Response: **Thank you for your observation. We have replaced the term 'solubilization' with 'dispersion' to refer to starch in water.

**Pp12. Flow profile analysis. Please, unify: “flowability index” or “flow index”.**

**Response: **Within the paragraph, we unified the term "flow index", thank you for the observation.

**Pp12. Two consecutive paragraphs begin with "Regarding the Flow index." Please review the wording.**

**Response: **Thank you for the suggestion; we took your recommendation into account and improved the writing throughout the text.

**Pp12. “dilettantish fluid” or “dilatant fluid”?**

**Response: **Thank you for the observation; we changed the term "diletent fluid" to the correct term "dilatant fluid".

**Pp13. Viscoelastic properties - Temperature sweep. Figure 5 needs more details. First, the color assigned to each module should be clarified in the figure caption. Second, it should be specified which curve corresponds to the heating phase and which to the cooling phase. Without this information, it is not possible to follow the discussion.**

**Response: **You are absolutely right, Figure 5 needs more details. Therefore, we have specified the color assigned to each modulus and clearly indicated which curve corresponds to both the heating phase and the cooling phase.

**Pp13. “progressive exudation” by “progressive lixiviation”.**

**Response: **You are absolutely right, the change was made using the correct term: progressive lixiviation. “There is a progressive lixiviation of amylose in the swollen granules, where gel-like linkages are formed. Researchers have reported the effect of amylose to increase the firmness of gels during cooling as one of the initial causes of gel firmness. Thus, during the cooling stage, the moduli stay constant with the predominance of the elastic modulus [59].”

**Pp 14. “where aw is less than 0.85”. Lower?**

**Response: **Thank you for the observation. The requested change has been made by using the term "below" instead of "lower," which improves the accuracy of the wording. The modified version is as follows:

 Table 2 shows that in foods such as sacha inchi flour and taro flour, where water activity is below 0.85, Salmonella spp. and other pathogenic bacteria, such as Staphylococcus, can survive in a viable but non-culturable state for extended periods due to their increased resistance to thermal processes. However, although microorganism growth is not sustained at low water activities, foodborne bacteria and fungi can easily contaminate the flour and survive for extended periods with a reduced reproduction rate [24].

**Pp 15. Conclusions. “The method that allows for the highest concentration of protein in sacha inchi is mechanical defatting with a protein result of 73%, compared to that obtained by the wet method, which resulted in 31%.” This does not belong in the Conclusion section, but rather in the Results and Discussion section.**

**Response: **You are right, this statement “The method that allows for the highest concentration of protein in sacha inchi is mechanical defatting with a protein result of 73%, compared to that obtained by the wet method, which resulted in 31%”  does not belong in the conclusions section, so we have taken your suggestion into account and have properly relocated it.

**Pp 15. “However, during the production of sacha flour with the mechanical method, bioactive compounds are lost, which is why lower values of total polyphenols and antioxidant activity are obtained, compared to taro flour.” In this sentence, methods for obtaining Sacha Inchi flour are compared, but it is compared with taro flour. Please review the wording.**

**Response: **Thank you for the observation. We have improved the wording based on the provided recommendation. “Mechanical defatting stood out as the most efficient method

 for obtaining high-protein flours from sacha inchi. However, this process was observed to reduce the preservation of bioactive compounds, decreasing antioxidant activity and total polyphenol content compared to the wet milling method.”

**Pp 15. “The viscosity profile evaluation determined that the starch contained in taro provides good viscosity characteristics, being this an important ingredient for the development of foods such as creams, pastes, sauces, among others.” This is useful for foods that require a heating process to allow starch gelatinization and hence viscosity development.**

**Response: **We took the suggestion into account and have modified it as follows: “The

 viscosity profile evaluation determined that the starch present in taro provides good viscosity characteristics, making it an important ingredient for the development of foods such as creams, pastes, sauces, among others. These properties are particularly useful in foods that require a heating process to allow starch gelatinization and, therefore, viscosity development. However, mixtures with an excess of taro have a higher rate of retrogradation, which represents a disadvantage for food stability during storage.”

**Pp15. “the optimum level of inclusion”. In fact, the optimal level will depend on the food that is being formulated.**

**Response: **You are absolutely right; the term used was not appropriate. Therefore, we have attached an improved version of the conclusion below: “Through the viscoelasticity analysis

 (temperature sweep), it was determined that an inclusion level of defatted sacha inchi flour between 25% and 50% results in a stable and consistent gel, which could be utilized in various food applications.”

**Pp16. “Although taro’s drying temperature of 60 °C maintains polyphenols and antioxidant activity”. We do not know if they "maintain," as it was not compared against other drying temperatures.**

**Response: **You are absolutely right; the term used was not appropriate. Therefore, we have attached an improved version of the conclusion below: “The drying temperature of taro at 60 °C preserves polyphenols and antioxidant activity at significant levels; however, it does not allow the inactivation of bacteria such as *Salmonella spp*., which has shown resistance to thermal treatment in recent years.”

**Pp16. “using gluten-free orphan raw materials”. What do authors mean by "orphan"?**

**Response: **We have replaced the term 'orphan' and improved the wording of this conclusion: “ The development of foods using gluten-free and underutilized raw materials, such as taro and sacha inchi, which easily adapt to regions facing food security challenges, represents a promising alternative for offering the market highly nutritious and gluten-free products.”

# Reviewer report: Reviewer response for version 3

**DOI:** 10.5256/f1000research.174336.r341939

**Published:** 2024-11-23

**Authors:** Elisa Julianti

**Affiliations:** 1 Department of Food Science Faculty of Agriculture, Universitas Sumatera Utara, Medan, North Sumatra, Indonesia

Thank you for the opportunity to review the revised article and the author responses. I have no further comments to make at this time.

Is the work clearly and accurately presented and does it cite the current literature?

Yes

If applicable, is the statistical analysis and its interpretation appropriate?

No

Are all the source data underlying the results available to ensure full reproducibility?

Yes

Is the study design appropriate and is the work technically sound?

Partly

Are the conclusions drawn adequately supported by the results?

Yes

Are sufficient details of methods and analysis provided to allow replication by others?

Partly

Reviewer Expertise:

Flour and Starch from Root and Tuber Crops as Alternatives to Wheat Flour

I confirm that I have read this submission and believe that I have an appropriate level of expertise to confirm that it is of an acceptable scientific standard.

# Reviewer report: Reviewer response for version 2

**DOI:** 10.5256/f1000research.155702.r317396

**Published:** 2024-09-17

**Authors:** Umi Purwandari

**Affiliations:** 1 University of Trunojoyo Madura, Madura, Indonesia

The paper was written carefully enough and data were analyzed thoroughly using recent related publication. However, microbiological analysis did not seem relevant to the work, and therefore it would be better deleted. 

 The term 'flour size' was confusing. Was it starch granule size, and if it was so, did the authors measure it, or is that any supporting argument for judging  the size. 

 Some parts were not correctly written, such as in page 10 where it was refer to Table2 for oil concentration. While Table 2 was about antioxidant activity. Also, statistical notation for Table 2, ABTS did not look right.

Is the work clearly and accurately presented and does it cite the current literature?

Partly

If applicable, is the statistical analysis and its interpretation appropriate?

Yes

Are all the source data underlying the results available to ensure full reproducibility?

Yes

Is the study design appropriate and is the work technically sound?

Yes

Are the conclusions drawn adequately supported by the results?

Yes

Are sufficient details of methods and analysis provided to allow replication by others?

Partly

Reviewer Expertise:

Food science, food microbiology

I confirm that I have read this submission and believe that I have an appropriate level of expertise to confirm that it is of an acceptable scientific standard, however I have significant reservations, as outlined above.

# Response

**Published:** 2024-10-31

**Authors:** Karen Muñoz

**Affiliations:** University of Cauca, Colombia

Dear Dr Purwandari, Thank you very much for your thoughtful and constructive feedback on our manuscript. We appreciate the time and effort you dedicated to reviewing our work, as well as your insightful suggestions, which have greatly helped us improve our paper. Below, we address each of your comments in detail:

 1.The paper was written carefully enough and data were analyzed thoroughly using

 recent related publication. However, microbiological analysis did not seem relevant

 to the work, and therefore it would be better deleted.

 Regarding the microbiological analysis, it is important to note that this was conducted to

 evaluate the initial state of the obtained flours concerning their microbiological

 composition. Since these flours are intended for food consumption, it is essential to know

 the level of microbial growth and to ensure that they fall within the permissible ranges

 established by current regulations governing such products.

 2. Some parts were not correctly written, such as in page 10 where it was refer to

 Table2 for oil concentration. While Table 2 was about antioxidant activity. Also,

 statistical notation for Table 2, ABTS did not look right.

 With the purpose of addressing the question raised, the information was supplemented

 using the correct term ;granulometry.; Additionally, the article mentions the following

 textually “Comparing SIF-DM flour with SIF-WM, the defatting method produces a

 flour with higher viscosity due to the interaction of water with proteins and starch,

 considering that SIF-DM has 72.62% protein. This result can be attributed to the fact

 that the lower viscosity of sacha inchi flour obtained by the wet method is due to the

 sample;s particle size (sieving with a standard mesh size of 425 to 600 microns), as it

 was not possible to reduce particle size due to its high fat content, and the resulting

 granules from the liquefaction and subsequent drying processes were used.

 Therefore, the endogenous lipids present in the taro flour form a type of protective

 layer around the aggregate of carbohydrates and proteins, preventing water

 absorption and the subsequent swelling of starch”.

 3 Some parts were not correctly written, such as in page 10 where it was refer to

 Table2 for oil concentration. While Table 2 was about antioxidant activity. Also,

 statistical notation for Table 2, ABTS did not look right.

 Regarding the suggested corrections, these have been properly adjusted, where Table 1 was

 referenced and the scientific notation was modified.

# Reviewer report: Reviewer response for version 2

**DOI:** 10.5256/f1000research.155702.r317400

**Published:** 2024-09-10

**Authors:** Elisa Julianti

**Affiliations:** 1 Department of Food Science Faculty of Agriculture, Universitas Sumatera Utara, Medan, North Sumatra, Indonesia

The subject of this manuscript (“Characterization of sacha inchi ( *Plukenetia volubilis*) and taro ( *Colocasia esculenta*) flours with potential application in the preparation of both gluten-free and high protein foods”) falls inside the general scope of the F1000 Research. The study presents an interesting exploration of sacha inchi and taro flours as alternatives in gluten-free and high-protein food formulations. The choice of materials is commendable due to their nutritional benefits and potential applications.  However, in my point of view, the paper does need some revision before indexing, as follows:

 1. In the conclusion of abstract, Based on the characteristics obtained, what type of food is suitable for the application of sacha inchi and taro flour? For example, would it be bread, pasta, biscuits, or other types of products?

 2. In the introduction, it should be justified what are the drawbacks of using sacha inchi and taro as single food ingredients? Why do you want to combine sacha inchi and taro flour as food ingredients?

 3. In the mathods : The method does not describe the analytical procedure for sacha inchi almonds. Was any treatment applied to the seeds before analysis?

 Is there a reason for using the ratio of SIF and TF?

 4. In the discussion :

     - It should be explained what causes the difference in protein content obtained through the wet method and the mechanical defatting method.

    - Sacha inchi flour obtained through the wet method is flour derived from the dried, water-insoluble components. The water-soluble carbohydrate components are certainly not included in SIF. However, why is the carbohydrate content in sacha inchi almonds lower than in SIF-DM? Additionally, why is the carbohydrate content in SIF-DM lower, even though only the fat is removed during its production? Please explain.

   - In SIF-DM, there is a release of phenolic compound due to involving pressure and temperature, but why is the FPP content in SIF-DM higher than in SIF-WM? Does the released FPP not get lost during the processing?

   - It would be more meaningful if the researcher could show the starch content of each sample with different SIF and TF ratios.

   - Based on Table 3, the aw for all three types of flour is <0.85, which should prevent the growth of microorganisms. How do you explain the presence of Salmonella and mold in your flour products?

 5. In conclusion : What is the recommended ratio of SIF (to TF ?

Is the work clearly and accurately presented and does it cite the current literature?

Yes

If applicable, is the statistical analysis and its interpretation appropriate?

No

Are all the source data underlying the results available to ensure full reproducibility?

Yes

Is the study design appropriate and is the work technically sound?

Partly

Are the conclusions drawn adequately supported by the results?

Yes

Are sufficient details of methods and analysis provided to allow replication by others?

Partly

Reviewer Expertise:

Flour and Starch from Root and Tuber Crops as Alternatives to Wheat Flour

I confirm that I have read this submission and believe that I have an appropriate level of expertise to confirm that it is of an acceptable scientific standard, however I have significant reservations, as outlined above.

# Response

**Published:** 2024-10-31

**Authors:** Karen Muñoz

**Affiliations:** University of Cauca, Colombia

Dear Dr Elisa,Thank you very much for your thoughtful and constructive feedback on our manuscript. We appreciate the time and effort you dedicated to reviewing our work, as well as your insightful suggestions, which have greatly helped us improve our paper. Below, we address each of your comments in detail:

 1. In conclusion of abstract, Based on the characteristics obtained, what type of food is suitable for the application of sacha inchi and taro flour? For example, would it be bread, pasta, biscuits, or other types of products?

 In response to Question 1, the conclusion of the document has been adjusted to mention that, based on the obtained characteristics, “the analyzed flours represent a raw material with great potential for the development of products such as protein snacks due to the protein contribution of sacha inchi, good expansion due to taro starch, and being naturally gluten-free for individuals with celiac disease”.

 2. In the introduction, it should be justified what are the drawbacks of using sacha inchi and taro as single food ingredients? Why do you want to combine sacha inchi and taro flour as food ingredients?

 In response to Question 2, the introduction has been supplemented with information about some drawbacks or limitations of both sacha inchi and taro. “Sacha inchi can be unstable due to its high concentration of unsaturated fatty acids, making it sensitive to oxidation. Additionally, sacha inchi has limiting amino acids, such as lysine and tryptophan, which may cause low digestibility. Likewise, this seed contains antinutritional factors that limit its use as a raw material in the development of new products. On the other hand, taro contains antinutrients such as oxalates, which can restrict its fresh consumption due to the irritation they cause in the throat and mouth. However, the extrusion process significantly reduces these issues while simultaneously enhancing nutritional properties, such as increased protein digestibility. Based on this, combining sacha inchi and taro is an excellent option to complement their nutritional properties, increasing nutritional value and improving the stability and texture of the final product”.

 3. In the methods : The method does not describe the analytical procedure for sacha inchi almonds. Was any treatment applied to the seeds before analysis? Is there a reason for using the ratio of SIF and TF?In relation to Question 3, in the section on methods used to obtain sacha inchi flour, additional information is provided about the process followed prior to the proximal characterization of the sacha inchi kernel. It is explained verbatim that “the sacha inchi seeds were received as white kernels, meaning without shell or husk. For the compositional analysis of the kernel, they were manually ground with a mortar to reduce their size, thus facilitating the relevant tests”. Furthermore, the reason for using the SIF and TF ratio lies in their high nutritional value (carbohydrates and protein) and the fact that both raw materials have had limited scientific and industrial advancement, presenting a significant opportunity to leverage their potential.

 4. 4. In the discussion :

 - It should be explained what causes the difference in protein content obtained through

 the wet method and the mechanical defatting method.

 To address this question, additional information has been included to explain the cause of

 differences in protein content obtained through the wet milling method compared to the mechanical defatting method. The document states verbatim: “It is important to note that the protein content obtained through the wet milling method is lower compared to the mechanical defatting method. This is because, during the wet milling process, a milky suspension is generated, which is then filtered to separate the insoluble extract (cake) from the water-soluble extract (slurry). During filtration, part of the protein may remain in the soluble extract, which explains the lower protein concentration in the cake. In this study, the proximate analysis was performed only on the insoluble extract”.

 - Sacha inchi flour obtained through the wet method is flour derived from the dried,

 water-insoluble components. The water-soluble carbohydrate components are certainly not

 included in SIF. However, why is the carbohydrate content in sacha inchi almonds lower

 than in SIF-DM? Additionally, why is the carbohydrate content in SIF-DM lower, even

 though only the fat is removed during its production? Please explain.

 Regarding the question, why is the carbohydrate content in sacha inchi kernels lower than

 in SIF-DM? Table 1 shows that the kernel contains 6.67% carbohydrates, while SIF-DM

 showed 3.04% carbohydrates. In relation to the question of why the carbohydrate content in

 SIF-DM is lower, despite the fact that only fat is removed during its production: as shown

 in the following image, it is important to mention that during the oil extraction process, not

 only fat is removed but also some solids such as proteins, minerals, and some

 carbohydrates. These components may become trapped in the oil or be removed with the

 byproduct generated during extraction. Additionally, it is likely that some soluble

 carbohydrates dissolve or are removed along with the oil during mechanical extraction.

 - In SIF-DM, there is a release of phenolic compounds due to involving pressure and

 temperature, but why is the FPP content in SIF-DM higher than in SIF-WM? Does the

 released FPP not get lost during the processing?

 Regarding the question of why the free total phenol content (FPP) in SIF-DM is higher than

 in SIF-WM, it is important to consider the differences in the extraction processes. During

 the wet milling process (SIF-WM), phenolic compounds, which are water-soluble, can

 migrate into the liquid solvent during the maceration stage, resulting in a significant loss of

 these bioactive compounds in the solvent and thus decreasing the concentration of phenols

 in the final flour. In contrast, the mechanical defatting process (SIF-DM), a more physical

 method that does not involve liquid solvents, results in much lower phenolic compound

 loss. Without a significant aqueous phase, the phenols remain largely within the solid

 matrix of the flour.

 - It would be more meaningful if the researcher could show the starch content of each

 sample with different SIF and TF ratios.

 To complement this information in the article, the following text has been added detailing the

 carbohydrate percentage and protein content in each mixture with different inclusion levels. “The study design was based on the type of flour and its inclusion levels in the mixture, taking protein content and carbohydrate percentage as the most relevant factors in each treatment. The following results were obtained: the 25% SIF-DM:75% TF mixture had 22.69% protein and 64.83%

 carbohydrates; the 50% SIF-DM:50% TF mixture showed 39.34% protein and 44.23%

 carbohydrates; and the 75% SIF-DM:25% TF mixture yielded 55.98% protein and 23.64%

 carbohydrates. Meanwhile, the mixtures with SIF-WM presented the following values: 25% SIF-

 WM:75% TF with 12.42% protein and 73.47% carbohydrates; 50% SIF-WM:50% TF with 18.80% protein and 61.52% carbohydrates; and finally, the 75% SIF-WM:25% TF mixture with 25.17% protein and 49.56% carbohydrates”.

 - Based on Table 3, the aw for all three types of flour is <0.85, which should prevent the

 growth of microorganisms. How do you explain the presence of *Salmonella *and mold in

 your flour products?

 As mentioned in Table 3, the analyzed flours exhibit low water activity, with values below 0.85.

 This level of water activity is not suitable for the growth of most microorganisms, as the available water is insufficient to sustain basic metabolic processes. However, while low water activity inhibits the active proliferation of microorganisms, it does not guarantee the total absence of pathogens. Spores of certain microorganisms and bacteria, such as *Salmonella*, can survive under adverse conditions, remaining viable for extended periods. Additionally, many pathogens possess persistence mechanisms that allow them to survive in hostile environments. In this case, *Salmonella *can form protective structures called biofilms, which enable microorganisms to adhere to surfaces

 and resist disinfection treatments. Moreover, these biofilms not only act as physical barriers but also increase the tolerance of microorganisms to physical processes such as drying, protecting bacterial cells from environmental stress.

 5. In conclusion : What is the recommended ratio of SIF to TF ?

 To address this question, the conclusions state that through the viscoelasticity analysis (temperature sweep), it was determined that the optimal inclusion level of defatted sacha inchi is between 25% and 50%, which allows for the formation of a stable and consistent gel. Complementing this conclusion, the 25% SIF-DM:75% TF mixture has 22.69% protein and 64.83% carbohydrates, while the 50% SIF-DM:50% TF mixture shows 39.34% protein and 44.23% carbohydrates. These values are relevant for the development of products such as extruded snacks, where the protein contribution from sacha inchi is crucial, and the expansion capability provided by taro starch allows both ingredients to complement each other effectively.

# Reviewer report: Reviewer response for version 1

**DOI:** 10.5256/f1000research.143149.r175167

**Published:** 2023-09-04

**Authors:** Diego Salazar

**Affiliations:** 1 Facultad de Ciencia e Ingeniería en Alimentos, Universidad Tecnica de Ambato, Ambato, Tungurahua, Ecuador; 2 Universidad Complutense de Madrid, Madrid, Community of Madrid, Spain

The work presented is interested in using unconventional sources, their characterization, and their nutritional components. However, the work requires management of scientific writing, the information presented needs to be revised, and the correct use of units of measurement is missed, for example, %, degrees Celsius, among others. On the other hand, the methodology needs to be more precise and allow a straightforward reading of what has been developed. Finally, the results must be rewritten, and consider that critical analysis and discussion of the results must be carried out, not only using the data from the tables and rewriting them.

Is the work clearly and accurately presented and does it cite the current literature?

No

If applicable, is the statistical analysis and its interpretation appropriate?

No

Are all the source data underlying the results available to ensure full reproducibility?

No

Is the study design appropriate and is the work technically sound?

Partly

Are the conclusions drawn adequately supported by the results?

Partly

Are sufficient details of methods and analysis provided to allow replication by others?

Partly

Reviewer Expertise:

Food Science and Technology. Research in new sources of foods, andean crops, legumes, tubers.

I confirm that I have read this submission and believe that I have an appropriate level of expertise to state that I do not consider it to be of an acceptable scientific standard, for reasons outlined above.

# Response

**Published:** 2023-09-05

**Authors:** Karen Muñoz

**Affiliations:** University of Cauca, Colombia

The units are adjusted and the methodology is clarified.

 We kindly ask you to specify in which paragraph it is necessary to adjust the analysis, since in each test a comparative and statistical analysis of the results is performed.

# Response

**Published:** 2024-03-04

**Authors:** Karen Muñoz

**Affiliations:** University of Cauca, Colombia

Thank you for your valuable comments.

 I would like to continue the process of evaluation and correction of the article, for this I ask for new corrections, in order to be able to make them.

 Best regards

# Reviewer report: Reviewer response for version 1

**DOI:** 10.5256/f1000research.143149.r195999

**Published:** 2023-09-04

**Authors:** Oluwaseun P Bamidele

**Affiliations:** 1 University of Venda, Thohoyandou, Limpopo, South Africa

I worked on the PDF format of this manuscript and there is a fundamental error in the design of this study. The work would have been good if the authors work on compositing the two raw materials. There is no scientific contribution with way the study is presented. Although the Sacha inchi is new but not Taro.

 The major challenge with the manuscript is the design. Compositing the root (Taro) and the Sacha at different ratios or percentages will make a difference in flour, which is rich in protein and other bioactive compounds. 

 Also, the manner in which the author reported the analyses is poor. Reporting on Sacha is not enough, using it in food preparation will be the best.

 I recommend that the authors should proofread their manuscript. The English is not good enough.

 Please find the annotated pdf with further comments here.

Is the work clearly and accurately presented and does it cite the current literature?

No

If applicable, is the statistical analysis and its interpretation appropriate?

No

Are all the source data underlying the results available to ensure full reproducibility?

Yes

Is the study design appropriate and is the work technically sound?

No

Are the conclusions drawn adequately supported by the results?

No

Are sufficient details of methods and analysis provided to allow replication by others?

Yes

Reviewer Expertise:

Food Chemistry, Food processing and preservation, Encapsulation of bioactive compounds, Functional foods, Gamma irradiation.

I confirm that I have read this submission and believe that I have an appropriate level of expertise to state that I do not consider it to be of an acceptable scientific standard, for reasons outlined above.

# Response

**Published:** 2023-09-05

**Authors:** Karen Muñoz

**Affiliations:** University of Cauca, Colombia

The corrections suggested in the pdf of the manuscript were adjusted.

 A characterization of the two raw materials taro and sacha inchi is made.

 This research was done in order to pave the way for new projects on the development of food based on vegetable matrices. 

 Mixtures with inclusion levels of 25, 50 and 75% of sacha inchi flour and taro 75, 50 and 25% were worked in order to evaluate the bioactive, rheological and proximal properties.

 We kindly request to specify the paragraphs or sentences that should be adjusted to improve the analysis.

 In addition, improvements were made in the English language.
